# Concepts Describing and Assessing Individuals’ Environmental Sustainability: An Integrative Review and Taxonomy

**DOI:** 10.3389/fpsyg.2021.770470

**Published:** 2022-01-05

**Authors:** Laura M. Wallnoefer, Petra Riefler

**Affiliations:** Department of Economics and Social Sciences, Institute of Marketing and Innovation, University of Natural Resources and Life Sciences, Vienna, Austria

**Keywords:** human-nature relationship, pro-environmental behavior (PEB), green consumption, sustainability, pro-environmental dispositions, interdisciplinary, behavior, measurement

## Abstract

The need to encourage individuals as active change agents for sustainability transitions has led researchers across disciplines to conceptualize over 70 constructs to assess relevant dispositions to environmental protection and green consumption behaviors. The generated knowledge is, however, fragmented by an unconsolidated set of constructs developed within parallel literature streams. We, hence, use an integrative review method to capture conceptual and operational similarities and distinctiveness of constructs across disciplines in the literature, attempting to unify the knowledge base. Thereby, we identify 34 conceptually distinct constructs (along with 38 synonyms and 76 scales) relevant for the thematic synthesis on individual-level constructs framing contributions to environmental measures and issues. We followingly propose a taxonomy, systemizing constructs based on their concept type(s) (e.g., value, attitude, behavior) and contextual scope(s) of the environmental challenge (e.g., product choice, household practice) addressed. We capture these dimensions in critically assessing relevant and salient conceptual and operational features. We thus create a consolidated picture of extant constructs capturing individual-level environmental sustainability by which we intend a three-fold contribution to the interdisciplinary field. First, the taxonomy and guiding framework for the choice of constructs should assist substantive researchers in identifying appropriate constructs of interest. Second, the systematic integration of (dis)similar concepts available in parallel literature streams should assist future endeavors aiming at integrating substantive findings with regard to antecedents, consequences, and other relevant variables. Finally, the taxonomy revealed that conceptualizations mainly scatter around specific combinations of types and scopes while others remain unaddressed. Based on our critical assessment of the relevant features and resulting taxonomy, we identify avenues for future research dedicated to (i) enhancing conceptual rigor and measurement quality in the field and (ii) introducing concepts addressing missing but potentially valuable combinations of types and scopes (e.g., antecedents capturing green consumption contexts). We conclude that researchers engaging in the proposed avenues with conceptual, methodological, or empirical contributions should consider four critical aspects to advance knowledge accumulation and prevent fragmentation in the interdisciplinary field. We thereby hope to pave the way for a collective, interdisciplinary knowledge base of concepts used to describe and assess individual’s pro-environmental dispositions and practices of green consumption.

## Graphical Abstract

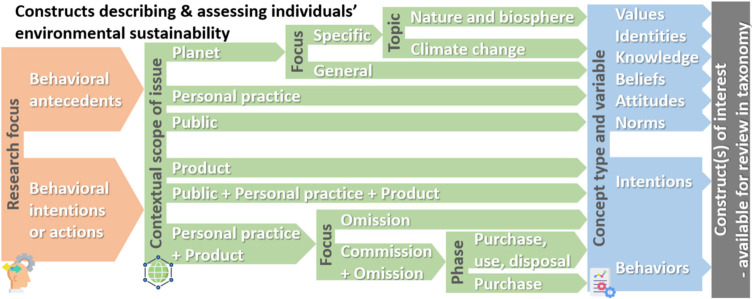

## Introduction

Changing individuals’ consumption patterns holds a considerable potential to mitigate climate change (Cinner, 2018; Nielsen et al., 2021). In their roles as consumers, investors, participants in organizations, members of communities, and citizens, individuals further affect both the supply and demand side of greenhouse gas (GHG) producing goods and services (Nielsen et al., 2021). The consumption from private households alone accounts for about 60% of the consumption-based GHGs and assumes 50–80% of the global use of land, material, and water (Ivanova et al., 2016). Efforts to reduce these emissions, however, continue to be insufficient in order to meet the 1.5° temperature rise target set in the Paris Agreement and, thus, demand more immediate and fundamental changes of individual and household behavior (IPCC, 2018). Thereby, understanding psycho-social factors or competencies which motivate individuals to change their consumption and lifestyle-related choices becomes a central task of psychological and educational research (Bamberg et al., 2021).

As a response, researchers from various backgrounds within and beyond the expanding breath environmental psychology engaged in the conceptualization of constructs to describe and assess individuals’ dispositions and behaviors in reference to a wide range of environmental challenges (for reviews see, e.g., Steg and Vlek, 2009; Schanes et al., 2016; Geiger et al., 2018; Trudel, 2018). As our interdisciplinary and integrative review will show, indeed more than 70 concepts have been introduced across disciplines to frame environmental sustainability from an individual’s perspective aiming to explain how and why individuals might adjust their activities for the benefit of the planet and future generations. Early examples include the belief of an ecological worldview, assessed with the new environmental paradigm (NEP) scale (Dunlap and Van Liere, 1978), and the general ecological behavior construct (Kaiser et al., 1999a). More recent examples include constructs focusing on climate change or the human-nature relationship specifically, i.e., climate change risk perception (van der Linden, 2015), and ecological identity (Walton and Jones, 2017), to name but a few. Behavioral constructs recently emphasize the importance of consumption reduction, i.e., environmentally oriented anti-consumption (EOA) (García-de-Frutos et al., 2018), and environmentally motivated consumption reduction (EMCR) (Lasarov et al., 2019). With a growing number of conceptualizations of relevant dispositions and behaviors, literature also witnesses an increase in substantive research incorporating these concepts to advance knowledge about their drivers, conditions, and consequences (for recent examples see Geiger et al., 2019; Zeiske et al., 2020; Welsch et al., 2021).

On the positive side, this particular attention to individuals and their role in sustainability transitions is topical and should provide relevant knowledge to foster green consumption for a sustainable development. On the negative side, however, the current use of numerous and unconsolidated concepts (and measurement scales) impedes knowledge integration about individuals as focal change agents. This range of concepts includes, for example, concepts capturing similar to identical conceptual domains despite carrying different construct names. At the same time, other constructs carry identical names while capturing divergent conceptual domains. To make things worse, numerous measurement scales considerably diverge from the underlying construct’s domain, endangering content validity by measuring something else than intended. From a research perspective, these and other problematic aspects make an informed choice from the plethora of available constructs and operationalizations difficult. From an institutional and managerial perspective, the lack of overview and knowledge accumulation limits their practical implementation to promote green consumption and further sustainability interventions and strategies. In short, both researchers and practitioners are currently confronted with a large number of potentially promising constructs for describing and assessing individuals’ pro-environmental dispositions and practices of green consumption but at the same time, lack guidance on how to handle them. An improved interdisciplinary and integrated understanding of *(ir)responsible* environmental dispositions and behaviors, however, is critical in the face of the increasingly complex nature of sustainability challenges resulting from consumption and lifestyle-related choices (Rodríguez-Casallas et al., 2020; Bamberg et al., 2021).

This review thus aims to provide an overview and systemization of extant constructs, developed across disciplines, describing and assessing concepts of individual-level environmental sustainability. For this purpose, we engage in an integrative review approach comprising three stages, i.e., (1) an interdisciplinary review of concepts and their key characteristics to reveal overlaps and differences, (2) the development of a taxonomy allocating these concepts based upon salient characteristics, i.e., their concept type and contextual scope of the framed environmental issue to facilitate the overview and guidance, and (3) a critical assessment of the concepts’ conceptual development and measurement instruments. In this way, our intended contribution to this interdisciplinary field is three-fold: First, the taxonomy should assist substantive researchers in identifying appropriate constructs to assess individual-level dispositions toward environmental sustainability and green consumption behaviors. Second, the systematic integration of (dis)similar concepts available in parallel disciplines should assist future endeavors aiming at integrating substantive findings with regard to antecedents, consequences, and other relevant variables. Finally, the critical assessment of the concepts’ conceptual development and operationalization identifies avenues for future research dedicated to enhancing conceptual rigor and measurement quality in the field.

Hereafter, we first describe our methodological approach to identify and synthesize relevant constructs for our review. Next, we introduce the taxonomy and critically review the allocated concepts with regard to theoretical, conceptual, and measurement aspects. We then highlight key issues from a conceptual perspective on constructs assessing individual-level environmental sustainability and discuss their implications for two proposed future research avenues and practice. We conclude with recommendations for further research.

## Methods

We applied an integrative review method, as this is acknowledged as particularly suitable for synthesizing knowledge across scientific communities within a growing research field (Snyder, 2019; Cronin and George, 2020). The method includes two main steps, namely (i) the literature review and (ii) the thematic synthesis (Cronin and George, 2020). Following Torraco (2005), we first conceptually structured the focal research domain to ensure our review contributes to a consolidated picture of the extant concepts. We, therefore, employed a preliminary literature scan focusing on research fields (i.e., environmental psychology, sustainability science, ecological economics, and marketing) and leading theories incorporating individual-level environmental sustainability concepts [i.e., Theory of Planned Behavior (TPB) (Ajzen, 1991), Value-Belief-Norm (VBN) Theory (Stern et al., 1999; Stern, 2000)]. Based on this scan, we derived and specified relevant literature streams and sources, other relevant theories, and conceptual domains for the (i) review and (ii) synthesis.

### Literature Review

**FIGURE 1:**
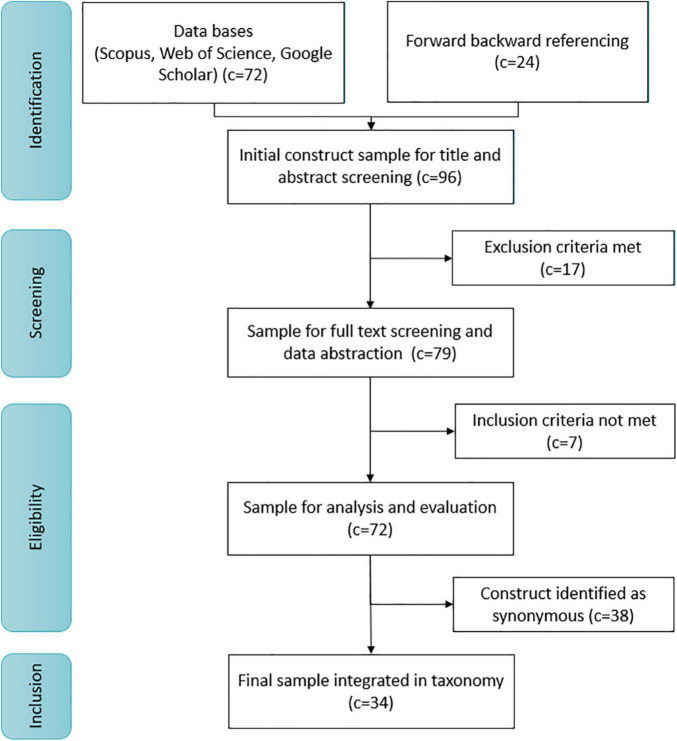
Concept search and selection strategy.

In the following step, we pursued the search strategy depicted in Figure 1 to identify relevant concepts. It indicates the number of constructs (c) identified, screened, defined as eligible, and finally included in the taxonomy.

We thus defined (i) search terms, (ii) information sources (e.g., databases), and (iii) eligibility criteria for articles and concepts. Concerning (i), we used a combination of frequently used terms referring to five aspects identified from the preliminary conceptual structuring (see Supplementary Appendix Table 1 for a list of these aspects, with search terms and their hierarchy in the relevant search strings). We then searched for articles with titles, abstracts, and keywords related to the relevant terms using relevant databases, namely *Scopus*, *Web of Science*, and *Google Scholar*. We complemented the databank search with a forward and backward search (Fischer et al., 2017b) based upon reference lists of identified papers and review articles (e.g., Geiger et al., 2018). We then refined the search to include only relevant subject areas derived from the preliminary literature scan. We exclusively considered peer-reviewed journal articles available in English that introduced new concepts, contributing to the conceptual development of existing concepts or developing measures to assess concepts, respectively. We furthermore regarded the quality of papers based on a combination of citation counts, journal ranking within the relevant subject areas, recency, and relevance (as employed by, e.g., Dowling et al., 2020). Purely empirical articles adopting concepts and measures were excluded from further screening. This search resulted in an initial set of 96 constructs describing or relating to individual-level environmental sustainability, with related articles found in the *Journal of Environmental Psychology* and *Environment and Behavior*, and *AMS Review*, to name but a few.

In the next step, we screened relevant articles’ sections on theory, construct definition, and operationalization to assess the eligibility of the initial set of 96 constructs. We, therefore, determined relevant inclusion and exclusion criteria at a concept level. A concept was *included* if (1) its conceptual domain relates to environmental sustainability as defined by Goodland (2017), *and* (2) it is based on a thorough conceptualization (MacKenzie, 2003) rather than being loosely introduced as an *ad hoc* term in a paper. Seven constructs failed to meet these criteria and were thus excluded from further consideration. Further, a concept was *excluded* if (1) it addresses a group-level rather than a personal-level phenomenon (O’Brien, 2018), *or* (2) it is used as an umbrella term rather than a distinct construct as defined by, e.g., MacKenzie (2003), MacInnis (2011), and Podsakoff et al. (2016). We thus excluded 17 constructs from further screening.

Having purified the set of concepts, we next identified concepts, sharing identical conceptual core, despite using different construct names, thus representing what we call “synonyms.” We, therefore, contrasted concept definitions and items of available measurement instruments across the 72 remaining constructs to identify identical or very close conceptual domains (see, e.g., MacKenzie, 2003; Podsakoff et al., 2016). We, for example, initially identified 12 concepts capturing the conceptual domain of human-nature relationships. By a first conceptual analysis, we identified groups of concepts capturing different facets of the relationship. By the second analysis of key features (i.e., theoretical background, conceptual dimensions, measurement items) and use of the constructs or terms within literature, we identified five specific conceptual cores, shared by 12 constructs. The conceptual core of, i.e., environmental self-identity (Van der Werff et al., 2013) is addressed by three further constructs, which are operationalized with similar to identical items and used as synonyms in the literature. By further examining citation patterns and temporal periods (Cronin and George, 2020), we found that the contribution of Van der Werff et al. (2013) first introduced the conceptual idea, and authors often referred to the paper to support their (synonymous) concept. Environmental self-identity (Van der Werff et al., 2013) thus presents what we distinguish as a core concept, stemming from a seminal paper that introduces a conceptual idea with a clear conceptual definition (Podsakoff et al., 2016), leading to subsequent research. Consequentially, this procedure revealed 38 synonymous constructs. In the taxonomy, such groups of synonymous concepts are represented by the more established, most referenced concept name (i.e., used in seminal papers), while the additional concept names are listed in Supplementary Appendix Table 2.^1^ Hence, the taxonomy focuses on a final set of 34 distinct concepts.

### Thematic Synthesis

In the last stage, we aimed at synthesizing the final set of 34 constructs to provide an integrative overview and guidance of extant conceptualizations. We allocated the 34 constructs to a taxonomy comprising of two dimensions, i.e., (1) concept type (based on definitions in Table 1) and (2) contextual scope (based on definitions in Table 2), which we identified as salient characteristics of concepts in this research domain. The first dimension represents latent theoretical individual-level variables describing the nature of the phenomenon to which the focal concept refers (see Podsakoff et al., 2016). The second dimension represents interdisciplinary perspectives taken on the role of individuals in contributing to environmental impacts and measures through their respective disposition(s) and behavior(s). This dimension integrates the views and approaches of the various disciplines regarding individual-level environmental sustainability, reflected by the different scopes of interest (i.e., an individual’s product choice, lifestyle, civic engagement, relation to nature). As Nielsen et al. (2021) stated, the appreciation of roles can bridge the gaps between different approaches and facilitate an interdisciplinary understanding of the concepts. We thereby respond to the need for more interdisciplinary and systems perspectives to understand the promotion behavioral changes, e.g., toward green consumption (Whitmarsh et al., 2021). The development of this taxonomy and the subsequent critical conceptual review, as in other synthesis processes (see, e.g., Sample et al., 2020), entailed an iterative process. For each construct, we derived the type and scope based upon their conceptual definition and, if available, operationalization (DeVellis, 2016), which yielded a preliminary taxonomy. Aiming at externally validating our construct allocation and dimensions defined for the thematic synthesis, we subsequently invited four senior researchers for an expert screening (adopted from Anderson and Gerbing, 1991). We provided them with a list of construct definitions and exemplary measurement items and asked them to assign each construct to (i) one (or more) concept type(s) and (ii) one (or more) contextual scope(s). The experts were able to allocate each construct to each of the suggested dimensions, thus confirming the applicability of the dimensions for the thematic synthesis of individual-level environmental sustainability concepts. This screening further confirmed the concept types as preliminarily derived. Concerning the contextual scope, expert meanings diverged from the preliminary allocation for two constructs. For these constructs, we re-evaluated the scopes based upon additional literature research and analysis. The expert screening thus yielded minor changes for the final taxonomy, which will be introduced and elaborated in detail in what follows.

**TABLE 1:** Dimension A: Conceptual definitions of salient conceptual types.

Concept	Definition
Values	“*(a) Concepts or beliefs, (b) about desirable end states or behaviors, (c) that transcend specific situations, (d) guide selection or evaluation of behavior and events, and (e) are ordered by relative importance*” (Schwartz and Bilsky, 1987, p. 551)
Identities	“*Relatively stable socially embedded meaning attached to the self that positions individuals within a web of socioecological relationships, based on shared personal characteristics, roles, and group memberships*” (Walton and Jones, 2017, p. 3)
Knowledge	“*The result of a person’s lifelong learning process, i.e., the voluntarily accessible and organized accumulation of veridical information (facts, rules, etc.)*” (Geiger et al., 2019, p. 2)
Beliefs	“*Understandings about the state of the world; they are facts as an individual perceives them*” (Dietz et al., 2005, p. 346)
Attitudes	“*A psychological tendency that is expressed by evaluating a particular entity with some degree of favor or disfavor*” (Eagly and Chaiken, 1993, p. 1)
Norms (subjective)	“*The perceived social pressure to perform or not to perform the behavior*” (Ajzen, 1991, p. 188)
Intentions	“*Intentions are assumed to capture the motivational factors that influence behavior, they are indications of how hard people are willing to try, of how much of an effort they are planning to exert, in order to perform a behavior*” (Ajzen, 1991, p. 181)
Behaviors	“*A highly specific single act*” (Heberlein, 1981, p. 21)

**TABLE 2:** Dimension B: Salient contextual scopes of environmental issues framed by concepts.

Context	Description
Planet	Concepts that address a planetary scope contextualize an individual’s contribution to environmental issues and measures from a global perspective. This perspective assumes a collective view of how individuals affect the environment on a global scale (Dunlap et al., 2000). Frequently addressed issues include the nature and environment in general, biophysical systems (i.e., ecological systems), climate change, and the conservation and preservation of natural resources.
Public	Concepts that address a public scope contextualize an individual’s contribution to environmental issues and measures from a civic engagement perspective. This perspective assumes a participatory view of how individuals affect the environment by their civic and political engagement (Alisat and Riemer, 2015). Examples include activism (demonstrations, advocacy) and non-activist support for the environmental movement (memberships, voting behavior, donations).
Personal practice	Concepts that address a personal practice scope contextualize an individual’s contribution to environmental issues and measures from a household perspective. This perspective assumes a lifestyle-driven view on how individuals affect the environment (Arnold et al., 2018). Frequently addressed issues are mobility, food, and shelter (Ivanova et al., 2016); and generally acting environmentally friendly within a private sphere.
Product	Concepts that address a product scope contextualize an individual’s contribution to environmental issues and measures from a perspective of product choice. This perspective assumes a consumption-specific view on how individuals affect the environment (Balderjahn et al., 2013). Frequently addressed issues include the purchase of green products, green consumerism, and the disposal of products.

## Overview of Taxonomy

**FIGURE 2:**
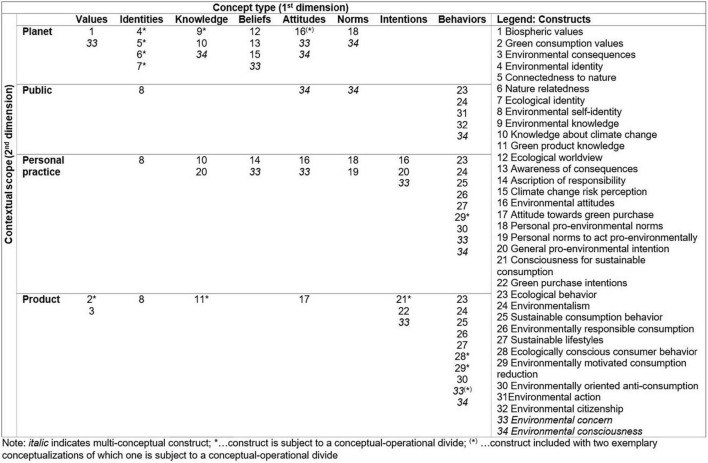
Taxonomy of constructs addressing individual-level environmental sustainability.

The 2-dimensional taxonomy (see Figure 2) includes 34 distinct individual-level concepts addressing environmental sustainability. We summarize the key features of each construct in Supplementary Table S1 to provide an overview. A guiding framework for the choice of the constructs of interest, based upon conceptual features such as the focal type of behavior (i.e., commission and/or omission) and consumption phase (i.e., purchase, use, disposal) can also be found in the Supplementary Figures S1, S2. Overall, the distribution of concepts within the taxonomy indicates a predominant focus on behavioral concepts within a personal practice and product scope. It further shows that the literature has introduced numerous concepts with a planet scope to describe individuals’ sustainability-related identities, knowledge, and beliefs. On the contrary, literature offers few or even no individual-level concepts for particular concept types (such as norms) and contexts (i.e., public). Given the nature of the included concepts, our taxonomy reveals that affective concepts received little attention in the reviewed literature compared to cognitive and conative variables.

In the following, we provide a detailed review regarding the conceptual development, identified synonyms, potential overlaps, and conceptual-operational divides of the 34 constructs. The following sections present the results of our thematic synthesis, structured by the concept types (i.e., columns of the taxonomy). Each section includes a detailed discussion of the constructs that refer to the same concept type and highlights their commonalities and differences regarding, e.g., the perspectives that are taken on the individual’s role and contextual scopes of the environmental issues addressed, as a result of the synthesis process.

### Values

“*Values function as standards by which actions, groups and individuals are evaluated.*” (Bouman et al., 2020, p. 1). The taxonomy includes three value concepts, i.e., biospheric values, environmental consequences, and green consumption values. While the former two relate to the planet scope, the latter addresses a product scope.

Biospheric values describe a “*concern with non-human species or the biosphere*” (Stern et al., 1993, p. 326) and are also referred to as biospheric personal values (Bouman et al., 2020). The construct, included in the VBN-Theory (Stern et al., 1999; Stern, 2000), bases upon the Norm-Activation Model (NAM) (Schwartz, 1977) and the Theory of Basic Human Values (Schwartz and Bilsky, 1987; Schwartz, 1992, 1994). Biospheric values, together with altruistic, egoistic, and hedonistic value constructs, are most relevant for environmental research (e.g., the prediction of environmental beliefs, preferences, and actions) (Stern et al., 1998; de Groot and Steg, 2008; Steg et al., 2014a). Accordingly, these four values are often jointly measured based on the Schwartz Value Survey (SVS) (Schwartz, 1994; Stern et al., 1998), typically using the Environmental Schwartz Value Survey (E-SVS) methodology (Steg et al., 2014b), or, most recently, the alternative Environmental-Portrait Value Questionnaire (E-PVQ) methodology (Bouman et al., 2018). Judging upon the face validity of the conceptual definition and operationalization of biospheric values, similarities to the concept of environmental values (Kaiser et al., 1999a,b), described as individuals’ social and moral values regarding the environment become apparent. However, the two concepts show different levels of specification. Biospheric values address a more general environmental context (with items such as “It is important to [him/her] to respect nature”; Bouman et al., 2018), whereas environmental values address topics such as animal rights and natural preservation more specifically (with items such as “I agree that animals should have legal rights”; Kaiser et al., 1999a,b).

The second value construct, i.e., green consumption values (GCV), is defined as a “*tendency to express the value of environmental protection through one’s purchases and consumption behaviors*” (Haws et al., 2014, p. 337). It was developed to measure green consumption values exclusively, as opposed to broader aspects such as environmental consciousness or attitudes toward socially responsible behavior in general (Haws et al., 2014). The conceptual development includes references to the Theory of Basic Values (Schwartz and Bilsky, 1987; Schwartz, 1992, 1994) and the Self-perception Theory (Bem, 1972). GCV overlap with the environmental dimension of the construct of sustainability-focused value orientation (Buerke et al., 2017). While per definition, GCV explicitly refer to values related to products, the operationalization with the GREEN-scale relates to additional aspects including personal practices (e.g., “I consider the potential environmental impact of my action when making many of my decisions”), identities (e.g., “I would describe myself as environmentally responsible”), and intentions (e.g., “I am willing to be inconvenienced in order to take actions that are more environmentally friendly”). As such, the operationalization of the construct is wider than its conceptual definition, which should be considered when applying the construct in empirical studies.

The product context is also addressed by the third value concept, i.e., environmental consequences, defined as “*concerns on how a product affects the environment, forest depletion, and energy usage in producing the product*” (Ramayah et al., 2010, p. 1421). The concept consequentially aims to assess an individual’s concern for various environmental issues related to the purchase, use, and disposal of products (e.g., air, water, and soil pollution). Being embedded within the Theory of Reasoned Action (TRA) (Fishbein and Ajzen, 1980), it thus contextualizes values with a clear focus on consumption, which renders the concept distinct from the above. To empirically assess environmental consequences, Ramayah et al. (2010) used a 3-item *ad hoc* measure.

In sum, all three value constructs address the importance individuals attribute to the environmental impacts of their activities within different contexts.

### Identities

Identities are ways of organizing information about the self (Clayton, 2003, in reference to Rosenberg, 1981). The proposed taxonomy includes five identity constructs, namely environmental identity, connectedness to nature, nature relatedness, ecological identity, and environmental self-identity. All five constructs cover different facets of the human-nature relationship (for a review, see, e.g., Balundë et al., 2019), which describe the connection and inclusion of nature as part of the self on the one hand, and the self-view as an environmentally friendly person on the other. While the former four constructs all address the planet scope, environmental self-identity indicates a personal practice context.

Literature of environmental psychology and environmental sociology understand the first identity concept in our taxonomy, i.e., environmental identity, in different ways. Social psychologists such as (Clayton, 2003) define environmental identity as:

[…] one part of the way in which people form their self-concept; a sense of connection to some parts of the non-human natural environment, based on history, emotional attachment, and/or similarity, that affects the way in which we perceive and act toward the world; a belief that the environment is important to us and an important part of who we are (Clayton, 2003, pp. 45–46).

Whereas environmental sociologists such as Stets and Biga (2003) conceptualize environmental identity as “*meanings that one attributes to the self as they relate to the environment*” (p. 406). The former conceptualization (Clayton, 2003) has received much attention throughout environmental psychology literature and has frequently been referred to by other conceptualizations of human-nature relationships (see, e.g., Mayer and Frantz, 2004; Nisbet et al., 2009; Brügger et al., 2011; Van der Werff et al., 2013; Walton and Jones, 2017). To measure environmental identity, Clayton (2003) developed the unidimensional environmental identity scale (EID). Reviews on the EID scale showed that it covers both facets of connecting to and identifying with nature (see, e.g., Olivos and Aragonés, 2011; Tam, 2013), thus characterizing the environmental identity concept as the broadest of the discussed identity constructs.

The second identity concept, i.e., connectedness to nature, is defined as “*individuals’ trait levels of feeling emotionally connected to the natural world*” (Mayer and Frantz, 2004, p. 503). It emphasizes the above-discussed aspect of individuals’ connecting to nature and seems widely established within the environmental psychology and sustainability literature. Still, literature also offers numerous other conceptualizations of this affective human-nature relationship. Examples include concepts of emotional affinity toward nature (Kals et al., 1999), the inclusion of nature in self (INS) (Schultz, 2002), implicit association with nature (Schultz et al., 2004), connectivity to nature (Dutcher et al., 2007), disposition to connect with nature (Brügger et al., 2011), and identification with nature (Schmitt et al., 2019). Against this background, a number of researchers have reviewed similarities and differences among these concepts (for details, see, e.g., Brügger et al., 2011; Balundë et al., 2019). With regard to measuring connectedness to nature, Mayer and Frantz (2004) provide the connectedness to nature scale (CNS) that addresses identities and attitudes within a planet scope. Similar to the EID-scale mentioned above, it overlaps with environmental concern rather than exclusively measuring its conceptual domain (Brügger et al., 2011). Literature also offers scales for the numerous other concepts listed above. On the positive side, Brügger et al. (2011) empirically demonstrate convergent validity of these scales, which means that all scales measure the same conceptual domain.

Turning to the third identity construct, nature relatedness is defined as “*individual levels of connectedness with the natural world*” (Nisbet et al., 2009, p. 718). It was developed to capture an experiential aspect of the human-nature relationship - in addition to the affective and cognitive aspect - which was seen as neglected by the connectedness to nature concept. The concept is operationalized by the 3-dimensional NR-Scale (Nisbet et al., 2009), which addresses more concept types (i.e., beliefs, behaviors) than included in the construct definition. Judging upon the scale’s face validity, two of the dimensions (labeled as NR-Self and NR-Experience) further overlap with Mayer and Frantz (2004) CNS. Recently, both concepts were summarized using the term “nature connection” (Mackay and Schmitt, 2019). In a later version of the NR-Scale, Nisbet and Zelenski (2013) drop the NR-Experience dimension from the scale. In this light, it seems that the NR-Scale might benefit from additional conceptual and psychometric work. Our review further shows that the NR-Scale is rather established in the health- and well-being literature, while the CNS has been frequently used in environmental psychology and sustainable consumption research (see Dong et al., 2020 for a recent study).

The fourth identity construct is the recently introduced ecological identity, defined as “*the extent and ways by which an individual views himself or herself as being a part of an integrated social and biophysical (i.e., ecological) system characterized by mutually beneficial processes and nested webs of relations*” (Walton and Jones, 2017, p. 10). The construct bases on both the Identity Theory and the Social Identity Theory (see Stets and Burke, 2000) with an elaborate conceptual development. It is specified as a 3-dimensional construct comprising aspects of *sameness*, *differentiation*, and *centrality* (for more details, see Walton and Jones, 2017), which are reflected in the ecological identity scale accordingly. However, the measurement instrument extends the focal planetary context in further addressing a product, personal practice, and public scopes.

The final identity concept is environmental self-identity, defined as “*the extent to which one sees oneself as a type of person whose actions are environmentally friendly*” (Van der Werff et al., 2013, p. 1258). Literature offers multiple synonymous concepts, such as pro-environmental self-identity (Dermody et al., 2018), environmentalist identity (Kashima et al., 2014), and green self-identity (Lalot et al., 2019). The concept overlaps with the *sameness* dimension of ecological identity (Walton and Jones, 2017), in regard to seeing oneself as an environmentally friendly person. Correspondingly, the measurement items of environmental self-identity relate to an individual’s identification with an environmentally friendly person (Van Der Werff et al., 2013). Judging upon both the concept definition and the items, the focal context seems vague and might be interpreted to stretch from product and personal practice to a public scope.

In sum, many of the reviewed identity concepts use different labels for measuring the same idea or conceptually overlap with each other. Connectedness to nature and nature relatedness both emphasize the affective and experiential facet of the human-nature relationship, while ecological identity and environmental self-identity focus on self-perceptions. The construct of environmental identity summarizes all these aspects in its conceptualization. Four of the five value constructs address a planet scope; however, their measurement instruments often include additional scopes, which requires careful consideration when used in substantive research.

### Knowledge

Individual’s knowledge about environmental issues is perceived as relevant cognitive variable driving pro-environmental behavior (see, e.g., Kollmuss and Agyeman, 2002; Bamberg and Möser, 2007). The proposed taxonomy includes three knowledge constructs, i.e., environmental knowledge, knowledge about climate change, and green product knowledge. Following the differentiation by Schahn and Holzer (1990), these constructs cover factual and abstract knowledge as well as action-related or concrete knowledge. The constructs differ in their contexts and measurement approaches. Environmental knowledge and knowledge about climate change measure knowledge about environmental topics (such as greenhouse gases and the energy transition), while green product knowledge specifically measures knowledge about the environmental impact of product use. Regarding measurement in general, knowledge can be measured as objective knowledge (i.e., correct answers to factual questions) (Geiger et al., 2019 refer to Cronbach, 1949) or as subjective knowledge (i.e., self-evaluation about personal level of know-how about a focal topic) (Geiger et al., 2019). Environmental knowledge and knowledge about climate change use objective, while green product knowledge uses subjective measures.

The concept of environmental knowledge has received considerable attention across the environmental psychology, business, and consumer studies literature (see, e.g., Lo and Fryxell, 2003; Frick et al., 2004; Chang and Wu, 2015; Geiger et al., 2019) and has been defined and conceptualized from different angles. Lo and Fryxell (2003) provide an inclusive definition covering both factual and action-related facets describing environmental knowledge as:

[…] a general knowledge of facts, concepts, and relationships concerning the natural environment and its major ecosystems. […] environmental knowledge involves what people know about the environment, key relationships leading to environmental aspects or impacts, an appreciation of “whole systems,” and collective responsibilities necessary for sustainable development (p. 48).

However, this definition is subject to an ongoing debate about the empirical differentiation of the concepts’ knowledge facets, despite the theoretical plausibility of the multifaceted structure of environmental knowledge (for a review, see Geiger et al., 2019). While Lo and Fryxell (2003) neglect to conceptualize the facets as separate dimensions, Frick et al. (2004) distinguish between dimensions of system-related (e.g., natural laws and ecological system), action-related (e.g., procedures aiming at environmental conservation), and effectiveness (i.e., of different environmentally friendly behaviors) knowledge. Accordingly, the available measurement instruments reflect the literature’s disagreement about the uni- or multi-dimensional nature of environmental knowledge. As such, operationalizations range from unidimensional scales (for examples see, e.g., Maloney et al., 1975; Kaiser et al., 1999b; Lo and Fryxell, 2003; Geiger et al., 2019) to three-dimensional scales (Frick et al., 2004). The unidimensional scales primarily relate to objective knowledge at the planet scope, whereas the multi-dimensional scales mainly embrace the product and personal practice scope.

Knowledge about climate change can be described as “*knowledge of causes and negative consequences of climate change*,” and is regarded as a “*cognitive aspect of risk judgments*” (Sundblad et al., 2007, p. 98). According to Leiserowitz et al. (2010), the construct can be divided into several general and overlapping categories, further described as “*knowledge about how the climate system works; specific knowledge about the causes, consequences, and potential solutions to global warming; contextual knowledge placing human-caused global warming in historical and geographic perspective; and practical knowledge that enables individual and collective action*” (p. 4). The concept is operationalized by three sub-scales measuring objective cause-, impact-, and response knowledge (van der Linden, 2015). While the above description suggests the construct relates to the planet and personal practice scopes, the measurement items appear to cover all four contextual scopes to varying degrees. The literature additionally offers the climate-related knowledge scale (Tobler et al., 2012b), which includes four subscales measuring knowledge about (i) physics, (ii) climate change and causes, (iii) expected consequences, and (iv) climate-related actions. Judging upon the face validity of both measurement instruments, Tobler et al. (2012a) address a more general knowledge level, while van der Linden (2015) provides more specific examples for the assessment.

The final construct in this section is green product knowledge, defined as “*subjective knowledge that is the consumers’ understanding of the environmental attributes and environmental impacts of green products*” (Wang et al., 2019, p. 2). It is theoretically based within the ABC-Theory (Guagnano et al., 1995) and is commonly used within sustainability and consumer research (for examples, see Liobikiene et al., 2016; Kumar et al., 2017). The construct partly overlaps with the concept of green product information (Ritter et al., 2015; Cheung and To, 2019). Both currently available operationalizations of green product knowledge (Kumar et al., 2017; Wang et al., 2019) use items adopted from extant measures. Kumar et al. (2017) adapted their operationalization from the recycling-centered measure of Ramayah et al. (2012), whereas Wang et al. (2019) employed the scales from Kanchanapibul et al. (2014) and Liobikiene et al. (2016), which focus on general ecological issues and the purchase and use of green products, respectively. Thus, these operationalizations fall short on covering the conceptual domain of green product knowledge within all consumption phases (purchase, use, and disposal) and relevant environmental consequences.

Summarizing, the three included knowledge constructs present different types of knowledge framing environmental issues spanning from planetary (such as mechanisms of climate change and ecosystems), over public related aspects, to the effectiveness of the own environmentally friendly actions. It can be observed that more recent conceptualizations put particular attention to contexts within one’s scope of action (e.g., product, personal practice, and public).

### Beliefs

Beliefs describe how individuals understand the world and depict facts as an individual perceives them (Dietz et al., 2005). As such, the first three constructs discussed in this section, i.e., ecological worldview, awareness of consequences (AC), and ascription of responsibility (AR), were introduced within the context of the VBN-Theory (Stern et al., 1999; Stern, 2000). The fourth concept, i.e., climate change risk perception (CCRP), was introduced more recently in the context of climate change (van der Linden, 2015).

The most established belief within the environmental psychology literature is that of an ecological worldview [also known as the New Environmental/Ecological Paradigm (NEP)]. The concept reflects “*fundamental views about nature and humans’ relationship to it”* focusing on “*[*…*] beliefs about humanity’s ability to upset the balance of nature, the existence of limits to growth for human societies, and humanity’s right to rule over the rest of nature*” (Dunlap et al., 2000, p. 427). It bases upon different social theories, challenging the dominant social paradigm (Pirages and Ehrlich, 1974). The ecological worldview is measured with the NEP-Scale, developed by Dunlap and Van Liere (1978) and later revised by Dunlap et al. (2000). The scale reflects five facets of an ecological worldview that address beliefs within a planet scope, e.g., about the fragility of nature’s balance. Although Dunlap et al. (2000) conceptualized the scale as unidimensional, they also discussed the possibility to treat it as multidimensional, depending on the context and population. This has led to the application of the NEP-Scale in a number of different contexts, which according to Stern et al. (1999), has rendered the NEP-Scale to one of the most applied social-psychology measures in the environmentalism literature. Indeed, it is frequently used to measure other concepts, such as environmental concern, values, and attitudes; see Dunlap (2008), Friías Armenta et al. (2010), and Hawcroft and Milfont (2010) for a review.

The second construct, i.e., the awareness of consequences (AC) of environmental conditions, is defined as “*key beliefs [*…*] that a particular condition has harmful consequences for other people*” (Stern et al., 1995b, p. 1614). Compared to the ecological worldview (Dunlap et al., 2000), it encompasses more specific consequences on a planet scope. As such, it addresses individuals’ anticipations of future environmental conditions for the self, for others, and the biosphere (Stern et al., 1995b). These three dimensions are also reflected in the construct’s operationalization by the general awareness of consequences (GAC) scale (Stern et al., 1995a). In detail, the scale’s *ACbio* dimension, which relates to the biosphere, partly overlaps with the concepts of societal consumer instrumentality awareness (Buerke et al., 2017), environmental beliefs (Kilbourne et al., 2002; Kilbourne and Pickett, 2008), as well as with the NEP-Scale (Dunlap et al., 2000). Concerning the latter, Stern et al. (1995b) conclude that the NEP-Scale and GAC-Scale are empirically similar in their relation to behavioral intentions, while they differ in their relation to values.

The third belief included is the ascription of responsibility (AR) for changing environmental conditions, defined as “*key beliefs [*…*] that the individual is responsible for those consequences in the sense that he or she can take action that would prevent them*” (Stern et al., 1995b, p. 1614). AR differs from the other constructs in this section, as it measures ascribed responsibility, which is a specific kind of belief directed toward one’s own action. A synonym to AR describing the same phenomenon is the concept of perceived responsibility for environmental damage (Peloza et al., 2013) operationalized by Wu and Yang (2018). Furthermore, AR conceptually overlaps with the concept of perceived consumer effectiveness (PCE) when applied within an environmental context (see Kim and Choi, 2005; Lee et al., 2014). In terms of AR measurement, Steg and Groot (2010) provide a scale that focuses on an individual’s feeling for *doing something to reduce problems*. In parallel, Wu and Yang (2018) adopt a scale from marketing literature (Peloza et al., 2013) and rather emphasize an individual’s feeling of *causing the problem*.

The final belief construct, i.e., climate change risk perception (CCRP), is described as “*a function of cognitive factors (i.e., knowledge about climate change), experiential processing (i.e., affective evaluations and personal experience) and socio-cultural influences (including social norms and broad value orientations)*” (van der Linden, 2015, p. 117). Its key dimensions relate to personal and societal risk judgments, respectively. Numerous authors provide numerous theoretical perspectives on risk perceptions in general as well as risk perceptions toward complex global issues, such as climate change, specifically. Examples include the Focus Theory of Normative Conduct (Cialdini et al., 1990) and the VBN-Theory (Stern et al., 1999; Stern, 2000). The construct conceptually and operationally overlaps with climate change belief (Brick et al., 2017), which assesses concerns and perceived risk of climate change. CCRP has received much attention across disciplines in both conceptual research (e.g., Lee et al., 2015; van der Linden, 2015) and substantive research relating to its role to engage individuals for climate change mitigation (e.g., Lacroix and Gifford, 2018). The measurement of CCRP (van der Linden, 2015) bases upon items developed by Bord et al. (2000) and Leiserowitz (2006), assessing the perceived likelihood of climate change events taking place in two dimensions (personal and global/societal). In addition, the literature offers scales measuring similar concepts of concern about climate change (Tobler et al., 2012a), concern for climate (Zhu et al., 2020), and climate concern (Alcock et al., 2017).

Overall, three of the four included beliefs share a planetary contextual scope with different degrees of specificity. As such, they assess an individual’s beliefs in an overall paradigm (NEP), the influence of humans in the environment (AC), and the likelihood of climate change (CCRP). In contrast to this societal perspective, the concept of the ascription of responsibility (AR) more specifically assesses beliefs concerning one’s responsibility for environmental issues. The review showed that current literature has not yet addressed such responsibility ascriptions specific to contexts of personal practices and product consumption, though this might be a relevant addition.

### Attitudes

Attitudes can be defined as “*the degree to which a person has a favorable or unfavorable evaluation or appraisal of the behavior in question*” (Ajzen, 1991, p. 188). The taxonomy comprises two attitudinal constructs, namely environmental attitudes and attitudes toward green products. While the former is an established construct within the environmental psychology literature and addresses the planet and personal practice scopes, the latter is a construct often used within the marketing literature and addresses the product scope.

The concept of environmental attitudes has received attention from numerous authors in parallel efforts (see, e.g., Kaiser et al., 1999b; Schultz et al., 2004; Milfont and Duckitt, 2004, 2010). This interest has resulted in two established approaches to model its attitude structure. One follows the more traditional three-component model conceptualizing cognitive, affective, and behavioral aspects as components of the attitudinal concept (see, e.g., Cottrell, 2003). The other approach perceives cognition, affect, and behavior as the bases from which the general evaluative summary of a particular psychological object derives, instead of being constituents of attitudes (Fabrigar et al., 2005). Based upon the first approach, Schultz et al. (2004) define environmental attitudes as “*the collection of beliefs, affect, and behavioral intentions a person holds regarding environmentally related activities or issues*” (p. 31). Based upon the second approach, Milfont (2007) refer to environmental attitudes as “*a psychological tendency that is expressed by evaluating perceptions of, or beliefs regarding the natural environment, including factors affecting its quality, with some degree of favor or disfavor*” (p. 12). The conceptualization of Milfont (2007) and Milfont and Duckitt (2010) bases on norm-related theories, while Schultz et al. (2004) neglect to refer to a specific theoretical foundation. While both definitions address the planet scope, Schultz et al. (2004) further include the personal practice context. Regarding the measurement, Schultz et al. (2004) borrowed items of four extant environmental measures, i.e., NEP-Scale (Dunlap et al., 2000), Environmental Motives Scale (EMS) (Schultz, 2001), a self-reported pro-environmental behavior scale (Schultz and Zelezny, 1998), and a revised version of Aron et al. (1992) Inclusion of Other in Self. Most recently, Kaiser et al. (2018) constructed a set of five specific-objectivity-based measures of environmental attitudes. Milfont and Duckitt (2010) developed a 12-dimensional environmental attitudes inventory (EIA), which covers beliefs, attitudes, and behaviors in reference to personal practices, publicly relevant actions, and planet-wide environmental issues. The operationalization thus seems broader than the underlying conceptual definition. Moreover, the dimensionality of the environmental attitudes concept has been intensively debated in the literature, with a tendency toward consensus for a multidimensional over a unidimensional conceptualization (see Milfont and Duckitt, 2010, for a summary). Further, the horizontal structure has been discussed and non-conclusively addressed in empirical research. As such, the EIA’s 12 dimensions have been modeled to load on a single second-order factor (i.e., Generalized Environmental Attitudes) or alternatively on two (correlated) second-order factors (i.e., preservation and utilization attitude) (Milfont and Duckitt, 2010; see also Kaiser et al., 2013 in a similar vein).

Turning to the second attitudinal concept, i.e., attitude toward green purchase, Chan (2001) neglects to provide an explicit conceptual definition. Instead, he refers to the definition of attitudes by Eagly and Chaiken (1993). The concept aims to capture individuals’ attitudes toward environmentally friendly products in specific. Hence, its conceptual domain is narrower than that of environmental attitudes discussed above. This narrow domain is also reflected in Chan (2001) three items-scale to assessing individuals’ (dis-)like of purchasing green products (based upon Taylor and Todd, 1995, global attitudinal measure).

Overall, this section demonstrates the variety of conceptualizations of individuals’ attitudes toward different environmental issues, dependent on the evaluated object. This is in line with the early findings on environmental attitudes by Heberlein (1981), who argued that having the environment as an attitudinal object is challenging, as it is difficult to define and can thus lead to different interpretations of the concept type and contextual scope.

### Norms

Norms are “*the perceived social pressure to perform or not to perform the behavior*” (Ajzen, 1991, p. 188). Our review shows that numerous norms are used to explain pro-environmental behaviors, even though some of these norms were originally conceptualized for studies on altruism (Schwartz, 1977) or behavior in general (Ajzen, 1991); for a review on personal norms, see Thogersen (2006). The taxonomy includes the two norms that specifically relate to environmental sustainability, which are personal pro-environmental norms (PPEN), and personal norms to act pro-environmentally. While the later construct specifically focuses on perceptions an individual holds toward own obligations, the former (PPEN) further includes perceptions on corporate and governmental obligations.

Personal pro-environmental norms (PPEN) are defined as “*the belief that the individual and other social actors have an obligation to alleviate environmental problems*” (Stern et al., 1999, p. 31) within the VBN-Theory (Stern et al., 1999; Stern, 2000). Other social actors include the government and businesses (Stern et al., 1999). The definition above does not allow to pinpoint the contextual scope as the focal actions individuals perceive as relevant to alleviate the environment are not defined. However, judging upon the face validity of the items included in Stern et al. (1999) unidimensional measure of PPEN, the normative actions touch a personal practice scope by addressing one’s own perceived obligation toward environmental protection (i.e., of tropical forests), as well as a planet scope by addressing the obligations of social actors. Conceptually, PPEN overlaps with the two constructs of ecological (Seyfang, 2005) and sustainable citizenship (Barry, 2006) from the environmental politics literature.

Personal norms to act pro-environmentally are defined as “*personal feeling of obligation to act pro-environmentally*” (Bouman et al., 2020, p. 4). The concept bases upon the personal norms concept, i.e., “*feelings of moral obligation to perform or refrain from specific actions*” as defined within the norm activation model (NAM) (Schwartz and Howard, 1981, p. 191). It conceptually overlaps with PPEN (Stern et al., 1999) regarding the norms individuals hold toward their own contributions to environmental problems and solutions. Literature fails to provide a dedicated personal norms scale. Instead, it borrows items from general personal norm measures (e.g., Steg et al., 2011; Van der Werff et al., 2013), which it relates to study-specific environmental actions [as, for example, household energy use (Steg et al., 2011; Bouman et al., 2020); participating in demonstrations (Steg and Groot, 2010); consuming sustainable products (Van der Werff et al., 2013); and water use (Verplanken and Roy, 2016)].

Summarizing, both included personal norms focus on the obligation of individuals to prevent harmful behaviors and conduct beneficial behaviors toward the environment. While both conceptual definitions fall short on delineating the context of these behaviors, the accompanying operationalizations reveal a contextualization along the spectrum of the product, personal practice, and public scopes. The PPEN measure specifies the environmental problems (i.e., climate change and loss of tropical forests) by contrast, the measures of personal norms to act pro-environmentally specify the particular behaviors.

### Intentions

Individuals’ intention to behave in particular ways depicts a central factor in both the TRA (Fishbein and Ajzen, 1980) and the TPB (Ajzen, 1985, 1991). In general, empirical research frequently measures intentions as a proxy for actual behavior. In the context of environmental sustainability, the review identified three concepts that can be classified into two types of intentional concepts, namely (a) intentions toward general behaviors (i.e., general pro-environmental personal intention), and (b) intentions toward specific purchase behaviors [i.e., consciousness for sustainable consumption (CSC) and green purchase intention (GPI)].

Regarding the first type, the construct of general pro-environmental personal intention is defined as “*[*…*] a more general intention to make efforts to protect the environment [*…*]”* (Lalot et al., 2019, p. 83). It is based on the TPB (Ajzen, 1985, 1991) as well as the Self-competition theory (Gollwitzer et al., 1982). While the above definition neglects to indicate a specific context, judging upon the face validity of Lalot et al. (2019) unidimensional scale, the focal intention refers to personal practices such as adopting more pro-environmental behaviors and decreasing “anti”-environmental behaviors.

Regarding the second type, CSC is defined as “*an intention to consume in a way that enhances the environmental, social and economic aspects of quality of life*” (Balderjahn et al., 2013, p. 182). Consequentially, it addresses not only environmental but also social and economic sustainability dimensions referring to the triple bottom line (Elkington, 1999). The construct was developed within the marketing literature, reflecting an intention within a product scope. In its operationalization, however, the CSC-Scale asks respondents to also rate statements such as “*I buy a product only if I believe that*…” and “*How important is it for you personally that*…,” thus additionally addressing behaviors and values. Along these lines, the authors note that the “*CSC model combines a consumer’s beliefs about the environmental (ENV), social (SOC) and economic (ECON) consequences of purchasing a product with the importance or personal concern the consumer attaches to these three consequences*” (Balderjahn et al., 2013, p. 184). Hence, it seems that the authors understand and operationalize CSC in a broader sense than indicated in the construct definition. Therefore, we highlight this three-fold scope accordingly – encompassing beliefs, values, and intentions – in our taxonomy (see Figure 2).

The construct GPI is defined as “*the likelihood that a consumer would buy a particular product resulting from his or her environmental needs*” (Chen and Chang, 2012, p. 507). It is derived from the classical “intention to buy” concept established in the marketing and retail literature. GPI differs from CSC by focusing exclusively on the product purchase rather than the whole product life cycle. The later introduced construct of purchase intention for environmentally sustainable products (PI) (Kumar et al., 2017) represents a similar conceptual core and thus a synonym to GPI. To measure GPI, several authors adapt classic “intention to buy” scales (see, e.g., Chan, 2001; Kanchanapibul et al., 2014; Sreen et al., 2018).

Summarizing, this section shows that the intentional concepts mainly address behaviors performed within a personal practice and product scope.

### Behaviors

In his review on environmental attitudes, Heberlein (1981) concludes that behaviors are highly specific, single acts. These acts are often specified as dependent variables in conceptual models [for example, in the TPB (Ajzen, 1985, 1991) or the VBN-Theory (Stern et al., 1999; Stern, 2000)]. Contextually, the 10 behavioral concepts included address actions or activities within the product, personal practice, and public scopes. Given the number of extant concepts and contexts, we further categorize the behavioral constructs into three groups based on the number of contextual scopes addressed. The first group addresses *all* scopes and includes two concepts, the second group encompasses six concepts addressing both within a product and personal practice scope, the third group comprises two concepts addressing the public scope exclusively. Regarding the first group, ecological behavior describes “*Actions which contribute toward environmental preservation and/or conservation*” (Axelrod and Lehman, 1993, p. 153 as cited by Kaiser et al. (1999a), p. 72). In the construct development, the authors mainly refer to the TPB (Ajzen, 1985, 1991). It overlaps with a number of other behaviors that reviewed for this taxonomy, i.e., goal-directed conservation behavior (Kaiser and Wilson, 2004), environmentally responsible behavior (Thøgersen, 2004), pro-environmental behavior (Bamberg and Möser, 2007), environmental behavior (Steg and Vlek, 2009) and environmentally friendly behavior (Liobikiene and Juknys, 2016). Among the numerous scales available to measure ecological and pro-environmental behavior, the Generalized Ecological Behavior (GEB) measure might be considered as the most established given its frequent use and its sound psychometric properties (for a review, see Lange and Dewitte, 2019). The GEB-Scale was originally developed by Kaiser (1998) and encompassed seven subscales, later, Kaiser and Wilson (2004) revised it into a measure with six subscales (i.e., energy conservation, mobility and transportation, waste avoidance, consumerism, recycling, and vicarious social behaviors toward conservation) reflecting different difficulty levels of enacting behaviors. The scale items strongly focus on the product and personal practice scope, while only a few items address the public scope. GEB-Scale items have also been used to assess other concepts such as environmental engagement (Kaiser and Byrka, 2011), ecological lifestyles (Arnold et al., 2018), environmental attitudes (Kaiser et al., 2007), and sustainable behavior (Corral-Verdugo et al., 2015). Consequentially, literature provides research on concepts with different labels but using identical measures. This situation results in an unconsolidated overall picture of substantive findings and leaves readers unclear on conceptual (dis)similarities among introduced concepts.

The second construct in the first group is environmentalism, “*defined behaviorally as the propensity to take actions with pro-environmental intent*” (Stern, 2000, p. 411). Environmentalism is part of the VBN-Theory (Stern et al., 1999; Stern, 2000) and is widely used in the literature. It is synonymous with the concepts of non-activist support for the environmental movement (Stern et al., 1999) as well as environmentally significant behavior (Stern, 2000). Environmentalism, by definition, describes not only a behavior but also an intention (i.e., a propensity to act). The specific context cannot be clearly derived from the definition, as the scope of the actions is not outlined. However, judging upon the face validity of the items (Stern, 2000), the three conceptual dimensions, i.e., consumer behavior, willingness to sacrifice, and environmental citizenship, cover behaviors in both a product and public scope as well as intentions within personal practice scope. As the intentions are not measured using propensity measures, a minor divide between conceptualization and operationalization becomes apparent. In contrast to the GEB-Scale, which primarily focuses on the product and personal practice scope, the environmentalism scale balances the number of items addressing the product, personal practice, and public scope. A more recent measure, with a similar balance of items, is provided with the pro-environmental behavior survey (Larson et al., 2015).

The behaviors in the second group of behavioral constructs can further be differentiated based on (a) the type of behavior addressed (i.e., commission behaviors and/or omission behaviors), and (b) the focal consumption phase (i.e., purchase, use, and/or disposing of products).

Three constructs in this group, i.e., sustainable consumption behavior (SCB), environmentally responsible consumption, and sustainable lifestyles, most comprehensively capture both commission and omission behavior within all three consumption phases. The constructs essentially differ in their conceptualization and the degree to which each consumption phase is addressed.

In detail, first, sustainable consumption behavior (SCB) is defined as “*individual acts of satisfying needs in different areas of life by acquiring, using and disposing goods and services that do not compromise the ecological and socio-economic conditions of all people (currently living or in the future) to satisfy their own needs*” (Geiger et al., 2018, p. 5). Sustainable consumer behavior (Trudel, 2018) is a synonymously used term. SCB is grounded in a number of theories and frameworks (including theories of planetary boundaries, ecological footprint, capability approach, and fundamental human needs, see Geiger et al., 2018) and includes socio-economic aspects in addition to the environmental aspects of sustainability. The construct has been operationalized by numerous authors (see, e.g., scales by Wang et al., 2014; Watkins et al., 2016; Fischer et al., 2017a). These measures, however, mainly capture the purchase and disposal phase whereas neglecting the sustainable use of products or services.

Second, environmentally responsible consumption is defined as “*any consumption-related behavior, namely, acquisition, use, and disposal, undertaken in a manner such that it reduces the negative impact of consumption on the environment*” (Gupta and Agrawal, 2018). The authors do not refer to any specific theory. In contrast to SCB, this concept does not include socio-economic aspects. The ten-dimensional scale (Gupta and Agrawal, 2018, p. 525) addresses the purchase, use, and disposal phase of products, and services in a more balanced way than SCB and sustainable lifestyles scales (as discussed below).

Third, the construct of sustainable lifestyles, defined as “*patterns of action and consumption, used by people to affiliate and differentiate themselves from others, which: meet basic needs, provide a better quality of life, minimize the use of natural resources and emissions of waste and pollutants over the lifestyle, and do not jeopardize the needs of future generations*” (CSD, 2004, p. 48) is conceptually similar to ecological lifestyles (Arnold et al., 2018) and green lifestyles (Lorenzen, 2012). It differs from the above concepts by (i) putting practices into focal interest and (ii) reflecting individuals’ potential use of behaviors to form identities. Accordingly, sustainable lifestyles are understood to encompass both pro-environmental behavior and a green self-image (Welsch et al., 2021). This specific focus is also reflected in the construct’s basis within the Theory of Actions and Habitus (Bourdieu and Nice, 1984) and the Pragmatists action theory (Giddens, 1991). However, the conceptual premise that lifestyles are identity-driven behaviors is not reflected in the currently available operationalizations of sustainable lifestyles (for examples, see Barr and Gilg, 2006; Gatersleben et al., 2010; Starcic et al., 2018), as the latter captures solely actual behaviors neglecting any identity aspect.

Fourth, the construct of ecologically conscious consumer behavior (ECCB) by Roberts and Bacon (1997), for which the authors define an ecologically conscious consumer as “*one who purchases (avoids) products and services which he or she perceives to have a positive (negative) impact on the environment*” (Roberts and Bacon, 1997, p. 84). Literature provides multiple conceptualizations of the construct, using terms such as green purchase behavior (Chan, 2001), green product consumer choice behavior (Lin and Huang, 2012), and responsible consumer behavior (Buerke et al., 2017). These concepts base on different theories, including Pirages and Ehrlich (1974) dominant social paradigm (Roberts and Bacon, 1997), Fishbein (1979) TRA (Chan, 2001), Sheth et al. (1991) theory of consumption values (Lin and Huang, 2012), and the Stern (2000) VBN-Theory (Buerke et al., 2017). Despite these differences, all these concepts share the same conceptual core and are thus summarized in the taxonomy (see Figure 2) under the umbrella term ECCB. To measure ECCB, Roberts and Bacon (1997) use a scale that not only addresses the product scope (as suggested by the above definition) but also the personal practice scope. The literature further provides scales for the previously mentioned synonyms (Chan, 2001; Lin and Huang, 2012; Buerke et al., 2017), which all exclusively assess purchase behavior within the product scope.

The final two behaviors in the second group, i.e., environmentally motivated consumption reduction (EMCR) and environmentally oriented anti-consumption (EOA) show a specific focus on the omission of harmful behavior to the environment, which differentiates them conceptually from the previously discussed concepts. The constructs differ among themselves in their focus on either reducing or rejecting consumption, respectively. EMCR is defined as “*the extent to which consumers lower their consumption in certain domains with the explicit intent to protect the environment*” (Lasarov et al., 2019, p. 282), while environmentally oriented anti-consumption (EOA) is defined as “*acts directed against any form of consumption, with the specific aim of protecting the environment*” (García-de-Frutos et al., 2018, p. 413). EMCR bases upon the motivated reasoning framework and sustainable consumption (see Lasarov et al., 2019), whereas EOA builds upon consumer resistance and anti-consumption manifestations (see Black and Cherrier, 2010; Cherrier et al., 2011; Chatzidakis and Lee, 2013). Contextually, EMCR addresses all three consumption phases on a personal practice and product scope, although using a unidimensional four-item scale in its operationalization. Judging upon face validity, it appears questionable whether these items can to fully reflect the underlying conceptual domain. Hence, construct validity should be addressed by future research. EOA has not yet been operationalized. Instead, a recent study uses behavioral measures from the five Eurobarometer studies on climate change (see Gesis-Leibniz Institute for the Social Sciences, 2020) to approximate EOA (Ortega Egea et al., 2020). These borrowed measures refer to avoidance behaviors such as the purchase of local products, car use, and short-haul flights, indicating a focus on the product and personal practice scope. Both EMCR (Lasarov et al., 2019) and EOA (García-de-Frutos et al., 2018) are rather recent behavioral constructs that focus on different levels of consumption reduction, ranging from a partial to a complete reduction of consumption. For establishing these concepts in future research, however, further methodological work including rigorous scale development (Netemeyer et al., 2012) appears warranted.

The third group of behavioral constructs, i.e., environmental actions and environmental citizenship, exclusively addresses (different levels of) public action. Environmental actions are defined as “*intentional and conscious civic behaviors that are focused on systemic causes of environmental problems and the promotion of environmental sustainability through collective efforts*” (Alisat and Riemer, 2015, p. 14). The construct conceptually and operationally overlaps with the environmental citizenship dimensions of the environmentalism concept (Stern, 2000). Environmental action is assessed with a two-dimensional scale, i.e., anticipatory action and leadership action. The items range from the assessment of low-level civic actions (such as educating oneself about environmental issues) to high-involvement political activism (such as organizing a protest) (Alisat and Riemer, 2015). The construct has been applied in conceptual and empirical research across the literature of environmental psychology, education, and sustainability within conceptual research (see, e.g., Riemer et al., 2016; Jia et al., 2017; Gkargkavouzi et al., 2019).

Environmental citizenship is defined as “*the engagement in political activities aimed at supporting environmental causes*” (Takahashi et al., 2017, p. 114). While the construct is often conceptualized as a dimension of pro-environmental behavior (see, e.g., Stern, 2000; Markle, 2013; Larson et al., 2015), a number of studies examine the concept of environmental citizenship individually (e.g., Steg et al., 2011; Schmitt et al., 2019; Song et al., 2019). Synonyms referring to the core idea of environmental citizenship comprise green citizenship (Dean, 2001), environmental citizenship behavior (Song et al., 2019), pro-environmental activist behavior (Schmitt et al., 2019), and environmental activism (Steg et al., 2011; Lee et al., 2019). Arguably, the concept overlaps with environmental action (Alisat and Riemer, 2015). The difference, however, can be seen in the type and level of individuals’ participation in environmental discourses and the perceived political pressure resulting from these actions (Alisat and Riemer, 2015). While environmental citizenship specifically addresses non-activist behaviors, environmental actions additionally include activism elements. To date, the literature fails to provide a well-developed scale for assessing environmental citizenship. Therefore, substantive research either borrows items from the environmentalism construct (Stern, 2000) or uses *ad hoc* measures capturing individuals’ activities such as donating, voting, or participating in demonstrations (Takahashi et al., 2017).

Given the plethora of similar concepts and problematic measures (including non-impactful behaviors and/or excluding conceptually relevant aspects), behavioral concepts to be used in substantive research should be selected with care. Interested readers are further referred to detailed reviews of behavioral concepts and measures by Markle (2013), Geiger et al. (2018), and Lange and Dewitte (2019).

### Multi-Conceptual Constructs

Finally, our taxonomy includes multi-conceptual constructs, which we define as constructs that encompass multiple concept types within their conceptual domain. This is the case for environmental concern and environmental consciousness.

An inclusive definition of environmental concern is provided by Fransson and Gärling (1999), who view the concept as ranging from “*a specific attitude toward environmentally relevant behavior to a more encompassing value orientation*” (p. 370). The authors theoretically ground the concept with reference to the NAM (Schwartz, 1977), the TPB (Ajzen, 1985, 1991), the VBN-Theory (Stern et al., 1999; Stern, 2000), and Self-Competition Theory (Gollwitzer et al., 1982). Social sciences show particular interest in environmental concern, which has led to the introduction of numerous concepts carrying the same label but basing upon diverging interpretations and conceptual domains (for reviews on conceptualizations and measurements see, i.e., Stern et al., 1995a; Fransson and Gärling, 1999; Dunlap and Jones, 2002; Rodríguez-Casallas et al., 2020). Overall, the concepts differ in two key aspects, i.e., (i) the specification of the relationship between environmental concern and environmental attitude, and (ii) the number of concept types included in the conceptual domain. Regarding (i), our review reveals three different interpretations, i.e., (1) environmental concern as an integral component of environmental attitudes (see Stern and Dietz, 1994; Bamberg, 2003; Schultz et al., 2004, 2005), (2) environmental attitudes as an affective component of environmental concern (see Dunlap and Jones, 2002), and (3) environmental concern as a synonym for environmental attitudes (Milfont and Duckitt, 2010).

To discuss (ii), we relate to two exemplary conceptualizations by Dunlap and Jones (2002) and Schultz et al. (2004). Schultz et al. (2004) define environmental concern as “*the affect (i.e., worry) associated with beliefs about environmental problems*” (p. 41), indicating a purely attitudinal nature of construct. In contrast, Dunlap and Jones (2002) define environmental concern as “*the degree to which people are aware of problems regarding the environment and support efforts to solve them and/or indicates a willingness to contribute personally to their solution*” (p. 485), indicating a four-fold concept type including attitudes, beliefs, intentions, and behaviors. Hence, both conceptualizations share the affective component, as one includes non-attitudinal aspects within the concept, and the other models them as related but distinct constructs.

With regard to measuring environmental concern, Schultz and colleagues use the environmental motives scale (EMS) (Schultz, 2001; Schultz et al., 2005), indicating a personal practice and planet scope. In contrast, Dunlap and colleagues measure cognitive, conative, and behavioral expressions of environmental concern with reference to all four scopes [e.g., banning products, green lifestyle behaviors, involvement in environmental movements, and worries about poor air quality; see Xiao and Dunlap (2007) and Xiao et al. (2013)]; for a review on measures of environmental concern see Rodríguez-Casallas et al. (2020). Both measures show overlaps with other, previously discussed operationalizations. The EMS (Schultz, 2001; Schultz et al., 2005) shares items with the EIA (Milfont and Duckitt, 2010) and the AC measure (Stern et al., 1995b). The measurement instrument of Xiao and Dunlap (2007) and Xiao et al. (2013) strongly relates to NEP-Scale. It is to note that the same applies to many other environmental concern measures in the literature (e.g., Bamberg, 2003; Kilbourne and Pickett, 2008; Marquart-Pyatt, 2012; Lee et al., 2014; Mohd Suki, 2016). In sum, there is not one established environmental concern scale, but a number of measures developed based on the environmental issue at the interest of the study (topic) and the way in which the concern is expressed [see Dunlap and Jones (2002) classification of environmental concern measures]. To accordingly reflect the different perspectives on environmental concern as reflected in literature, our taxonomy assigns environmental concern to values, beliefs, attitudes, intentions, and behaviors within multiple contextual scopes.

The second multi-conceptual construct, i.e., environmental consciousness, stems from the marketing literature and describes a “*multi-dimensional construct, consisting of cognitive, attitudinal and behavioral components*” (Schlegelmilch et al., 1996, p. 41). This description indicates that the construct comprises three conceptual types, i.e., knowledge, attitude, and behavior. The context is not clearly indicated in the conceptual definition but may be derived from the construct’s measure. The three dimensions are assessed through five positively correlated sub-scales (see Bohlen et al., 1993; Diamantopoulos et al., 2003). One sub-scale measures (subjective) knowledge of selected environmental problems within a planet scope. A second sub-scale measures individuals’ concerns about environmental quality using items that address attitudes, beliefs, and norms within a public and planet scope. Finally, the behavioral dimension is measured by three sub-scales capturing the level of environmentally sensitive behavior in a product, personal practice, and public scope, respectively. We indicate these derived contexts in the taxonomy (Figure 2).

In sum, the aspect of concern is central to both multi-conceptual constructs. From a conceptual perspective, beliefs and attitudes seem to be a focal domain to assess individuals’ awareness of environmental issues within all four contexts and their expected consequences for the self, others, and nature.

## Discussion

In this paper, we used an integrative review approach to synthesize the knowledge about over 70 extant concepts developed within different literature streams to describe and assess individual’s pro-environmental dispositions and practices of green consumption. As a result, we developed a two-dimensional taxonomy that synthesizes a set of 34 distinct concepts based on identical conceptual domains, conceptual overlaps, and multiple conceptualizations, identified through a critical assessment of the concepts’ conceptual development and operationalization. Through this knowledge synthesis, we aim to facilitate the overview and systematization of a so far unconsolidated set of conceptualizations capturing individual-level environmental sustainability and followingly contribute to the interdisciplinary research field in three ways.

As a first contribution, the insight provided on conceptual definitions, measurement instruments, and synonymously used concepts in combination with our guiding framework can assist substantive researchers in identifying appropriate constructs of interest. As a second contribution, the systematic integration of (dis)similar concepts, often developed parallelly within different streams of literature, can assist future endeavors, such as meta-analyses, aiming at integrating substantive findings with regard to antecedents, consequences, and other relevant variables. Based on our review, we recognize the need for future reviews dedicated to providing an additional overview of empirical findings concerning the focal concepts in our taxonomy and their nomological networks. Such an overview would assist stakeholders in their efforts to foster environmental sustainability at an individual level and at the same time benefit the research field by identifying directions for additional empirical research. The integration of the focal concepts into a consolidated picture, see Figure 2, further revealed extant conceptualizations to center around specific combinations of concept types and contextual scopes, framing particular environmental challenges. These refer to (a) broad and abstract environmental challenges (such as global warming or natural degradation), conceptualized by behavior-distal psychological concepts such as values, identities, knowledge, beliefs, and attitudes, and (b) narrow and practical routines related to daily activities and the purchase, use, and disposal of products conceptualized as individual norms, intentions, and behaviors. This gap between the contextual scopes of behavioral-distal and behavioral-proximal concepts could indicate the different views on an individual’s roles for environmental measures and challenges. It thus opens the ground for future research to investigate whether this gap, for example, impacts the use of behavioral-distal constructs as behavioral antecedents and relates to the value-action gap (Kollmuss and Agyeman, 2002). Other combinations of context types and contextual scopes than the ones referred to above seemingly received less or no attention in the literature. These “blind” spots in our taxonomy can provide a starting point for the introduction of relevant constructs. In contrast, the “crowded” spots can be the basis for research dedicated to enhancing conceptual rigor and measurement quality. As a third contribution, we thus discuss four critical aspects for the prevention of knowledge fragmentation, which we derived from the assessment of the constructs’ conceptual development and measurement instruments, to assist researchers who engage in the proposed research avenues. These aspects regard (i) the state of the conceptual development of particular constructs; (ii) the lack of discriminant validity among concepts developed in parallel efforts; (iii) the existence of conceptually distinct concepts carrying identical or similar names; and (iv) measurement instruments that appear inconsistent with concepts’ underlying conceptual domains; which we elaborate in the following.

The conceptual development of the focal constructs ranges from concepts that have gone through thorough development processes [e.g., biospheric values (Stern et al., 1999)] to concepts with scant development in reference to underlying theories and conceptual domains [e.g., attitude toward green purchase (Chan, 2001)]. Both theory and domain do, however, provide the basis for the subsequent construct development and operationalization (MacKenzie, 2003; Netemeyer et al., 2012). The lack of a theoretical foundation and/or a precise conceptual definition is thus problematic for multiple reasons (Podsakoff et al., 2016), which relate to the remaining three critical aspects. Consequentially, many of the revealed shortcomings that we reiterate in the following originate from this neglect at the very beginning of construct development^2^.

**FIGURE 3:**
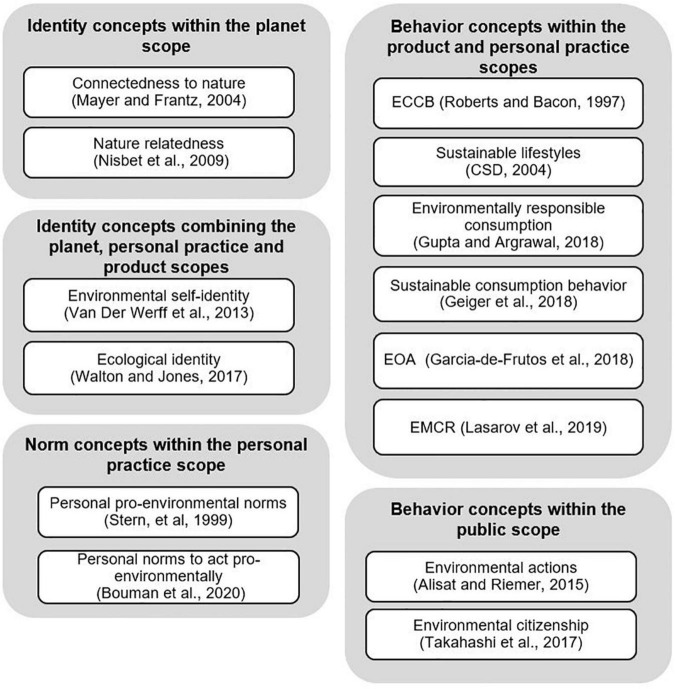
Overlapping conceptual domains.

Firstly, it endangers the establishment of discriminant validity toward related constructs. We thus recommend future research aiming to introduce additional concepts, to explicitly delineate them at a conceptual level from extant concepts (i.e., behavioral concepts within the product and personal practice scopes, see Figure 3) for which our taxonomy provides an overview, before engaging in the development process. This process should include tests of discriminant and convergent validity as proposed by scale development literature (DeVellis, 2016). For existing concepts, we encourage empirical research to examine discriminant validity among similar or overlapping concepts to better understand their similarities and distinctiveness (MacKenzie, 2003). This understanding would facilitate an informed choice of concepts for substantive research and contribute to developing an integrated knowledge base.

**FIGURE 4:**
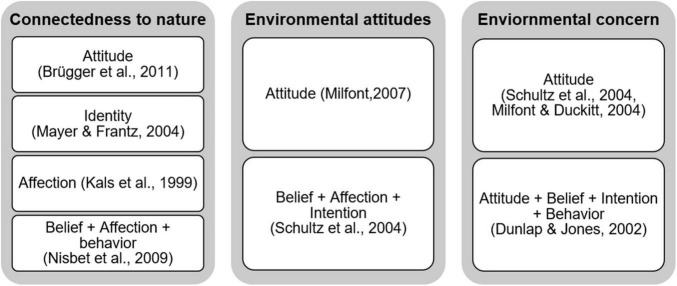
Concepts with multiple and inconsistent conceptualizations.

Secondly, it prepares the ground for conflicting interpretations of the concept’s meaning. Literature would thus benefit from additional research aiming at theoretically delineating divergent conceptualizations for the relevant concepts in our taxonomy (i.e., connectedness to nature, environmental attitudes, and environmental concern, see Figure 4). This endeavor would require theoretical and conceptual work on the one hand and empirical research to establish discriminant or divergent validity on the other hand (MacKenzie, 2003; Netemeyer et al., 2012). Such efforts might be complemented by empirical studies aiming to examine the concepts’ relative and/or complementary predictive power (MacKenzie, 2003) for relevant outcome variables. These insights would assist the advancement of the field by providing a clear picture of the unique contribution of these concepts and their adequate use in empirical models.

Thirdly, it jeopardizes the development of sound measurement scales (DeVellis, 2016). To prevent conceptual ambiguity and measurement misspecification, we urge future research to carefully delineate the type of construct for the development and/or adaption of scales from previous research when introducing new concepts (see MacKenzie, 2003). Following established guidelines, it is essential to provide a precise definition of the conceptual domain(s) and framed environmental problem(s) to ensure that measures closely reflect the conceptual meaning of the construct. This definition should consequentially build the basis for the process of scale development. Referring to extant concepts suffering from a conceptualization–operationalization divide, see Figure 2, further methodological work aiming at strengthening construct validity would be welcome. As such, one might need to engage in fresh scale development basing upon the extant conceptual definition, tightly following scale development guidelines, and engaging in rigorous scale validation (De Vellis, 2003). This methodological work is crucial as the validity of empirical findings is directly affected by the quality (i.e., reliability and validity) of the used measures. The indicated divides further hint at the fact that the scale development process and reporting of psychometric properties for reviewed concepts are, in some cases, limited. A detailed review of the identified 76 scales available to measure the 34 concepts would thus be valuable to enhance measurement quality and consequently empirical validity in the research field.

In emphasizing the relevance of knowledge integration by considering the critical aspects introduced, we aim to foster conceptual advances in this interdisciplinary field, relevant for sustainable transitions. These advances can be critical to both academics and practitioners, as they can facilitate connecting members of knowledge communities and further foster knowledge diffusion and sharing (MacInnis, 2011). Our taxonomy revealed potential entry points for such advances, for example, by indicating that most of the behavioral constructs address a product or personal practice scope, respectively. The public and planet scopes, however, become relevant when considering the role consumers play as activists and global citizens to combat the increasingly pressing environmental challenges (Thiermann and Sheate, 2020; Bamberg et al., 2021; Nielsen et al., 2021). In contrast, most behavioral antecedents capture the role of individuals for relevant challenges from a more collective, global perspective. An evidence-based design of green consumption interventions thus demands a more integrated understanding of environmental dispositions and behaviors that drive agency toward environmental sustainability. This can be facilitated by the integration of knowledge about relevant concepts (and measurement scales), i.e., values, beliefs, and behaviors, which we attempted with this interdisciplinary overview, review, and synthesis of more than 70 extant concepts. We thus hope to encourage future research and reviews that bridge and unify the domain knowledge on individual-level concepts relevant to fostering green consumption practices.

## Author Contributions

LW contributed to the conceptualization, methodology, investigation, and writing – original draft. PR contributed to the conceptualization, methodology, writing – review and editing, and supervision. Both authors contributed to the article and approved the submitted version.

## Conflict of Interest

The authors declare that the research was conducted in the absence of any commercial or financial relationships that could be construed as a potential conflict of interest.

## Publisher’s Note

All claims expressed in this article are solely those of the authors and do not necessarily represent those of their affiliated organizations, or those of the publisher, the editors and the reviewers. Any product that may be evaluated in this article, or claim that may be made by its manufacturer, is not guaranteed or endorsed by the publisher.

## Footnotes

1 Further details will be discussed later in the following chapters. An overview of the synonyms for the relevant constructs, construct definitions, and measurement instruments are provided in the Supplementary Table S1. A list of the initial set of constructs is provided by the authors upon request.

2 We additionally provide a section that includes more detailed guidance with examples using the constructs of our taxonomy in the supplementary material dedicated to interested readers (p. 15).
