# Supplementary material for: Concepts Describing and Assessing Individuals’ Environmental Sustainability: An Integrative Review and Taxonomy
Source: Front Psychol. 2022 Jan 5;12:770470. doi: 10.3389/fpsyg.2021.770470 (PMC8766306; doi:10.3389/fpsyg.2021.770470)
Supplement: Supplementary file 1 [file Data_Sheet_1.pdf]

Concepts describing and assessing individuals' environmental sustainability: An integrative review and taxonomy

Supplementary Material

List of definitions

Table S1

List of conceptual definitions of constructs included in the taxonomy

Note: We added information on the concept types and contextual scopes indicated in the operationalization in the respective column using "Items:" for construct subject to a conceptual-operational divide, i.e. if concept type(s) and/or contextual scope(s) indicated in the definition diverged from those indicated by the operationalization of a construct.

Construct	Definition	Concept type	Contextual scope	Synonyms
**1 Biospheric values**	"*concern with nonhuman species or the biosphere"* (Stern et al., 1993, p. 326)	Value	Planet	Biospheric personal values (Bouman et al., 2020)
**2 Green consumer value**	*"tendency to express the value of environmental protection through one's purchases and consumption behaviors*" (Haws et al., 2014, p. 337).	Value<br>Items: Value, intention, identity	Product<br>Items: planet, personal practice, public	-
**3 Environmental consequences**	"e*nvironmental consequences can be measured as concerns on how a product affects the environment, forest depletion, and energy usage in producing the product*" (Ramayah et al., 2010, p. 1421).	Value	Product	-
**4 Environmental identity**	"*one part of the way in which people form their self-concept; a sense of connection to some parts of the nonhuman natural environment, based on history, emotional attachment, and/or similarity, that affects the way in which we perceive and act towards the world; a belief that the environment is important to us and an important part of who we are"* (Clayton, 2003, pp. 45–46)	Identity	Planet, practice<br>Items: planet, public, personal practice	-

**5 Connectedness to nature**	*"individuals' trait levels of feeling emotionally connected to the natural world"* (Mayer and Frantz, 2004, p. 503)	Identity<br>Items: identity, attitude	Planet	Emotional affinity towards nature (Kals, Schumacher,& Montada, 1999)<br>Inclusion of nature in self (INS) (Schultz, 2002)<br>Connectivity to nature (Dutcher et al., 2007)<br>Identification with nature (Schmitt et al., 2019)
**6 Nature relatedness**	"*individual levels of connectedness with the natural world"* (Nisbet et al., 2009, p. 718).	Identity<br>Items: identity, beliefs, behaviors	Planet<br>Items: planet, personal practice	-
**7 Ecological Identity**	*"the extent and ways by which an individual views himself or herself as being a part of an integrated social and biophysical (i.e., ecological) system characterized by mutually beneficial processes and nested webs of relations"* (Walton and Jones, 2017, p. 10).	Identity	Planet<br>Items: product, personal practice, public, planet	-
**8 Environmental self-identity**	*"the extent to which one sees oneself as a type of person whose actions are environmentally-friendly"* (Van der Werff et al., 2013, p. 1258).	Identity	Personal practice – without excluding public and products	Pro-environmental self-identity (Dermody et al., 2018)<br>Environmentalist identity (Kashima et al., 2014)<br>Green self-identity (Lalot et al., 2019)
**9 Environmental knowledge**	"*a general knowledge of facts, concepts, and relationships concerning the natural environment and its major ecosystems. […] environmental knowledge involves what people know about the environment, key relationships leading to environmental aspects or impacts, an appreciation of "whole systems", and collective*	Knowledge	Planet	-

	responsibilities necessary for sustainable development" (Lo and Fryxell, 2003, p. 48)			
**10 Knowledge about climate change**	*"knowledge about how the climate system works; specific knowledge about the causes, consequences, and potential solutions to global warming; contextual knowledge placing human-caused global warming in historical and geographic perspective; and practical knowledge that enables individual and collective action"* (Leiserowitz et al., 2010, p. 4)	Knowledge	Personal practice, planet	-
**11 Green product knowledge**	"*subjective knowledge that is the consumers' understanding of the environmental attributes and environmental impacts of green products"* (Wang et al., 2019, p. 2)	Knowledge	Product	
**12 Ecological worldview**	*"fundamental views about nature and humans' relationship to it"* focusing on " […] *beliefs about humanity's ability to upset the balance of nature, the existence of limits to growth for human societies, and humanity's right to rule over the rest of nature"* (Dunlap et al., 2000, p. 427)	Belief	Planet	New environmental paradigm (NEP) (Dunlap and Van Liere, 1978)<br>New Ecological Paradigm (Dunlap et al., 2000)
**13 Awareness of consequences**	*"key beliefs […] that a particular condition has harmful consequences for other people (or, in the extended version, for valued objects)"* (Stern et al., 1995, p. 1614)	Belief	Planet	-
**14 Ascription of responsibility**	"*key beliefs […] that the individual is responsible for those consequences in the sense that he or she can take action that would prevent them"* (Stern et al., 1995, p. 1614).	Belief	Personal practice	Perceived responsibility for environmental damage (Peloza et al., 2013)
**15 Climate change risk perception**	*"a function of cognitive factors (i.e., knowledge about climate change), experiential processing (i.e., affective evaluations and personal experience) and socio-cultural influences (including social norms and broad value orientations)"* (van der Linden, 2015, p. 117)	Belief	Planet	Climate change belief (Brick, Sherman, & Kim, 2017)<br>Concern about climate change (Tobler et al., 2012)<br>Climate concern (Alcock et al., 2017)

				Concern for climate change (Zhu et al., 2020)
**16a Environmental attitude**	*"a psychological tendency that is expressed by evaluating perceptions of or beliefs regarding the natural environment, including factors affecting its quality, with some degree of favour or disfavor"* (Milfont, 2007, p. 12).	Attitude Items: beliefs, attitudes, behaviors	Planet Items: personal practice, public, planet	-
**16b Environmental attitude**	"*the collection of beliefs, affect, and behavioural intentions a person holds regarding environmentally related activities or issues"* (Schultz et al., 2004, p. 31)	Beliefs, attitudes, intentions	Planet, personal practice	Pro-environmental attitude (Lavelle et al., 2015)
**17 Attitude towards green purchase**	Reference to definition of attitudes by (Eagly and Chaiken, 1993, p. 1): "*a psychological tendency that is expressed by evaluating a particular entity with some degree of favor or disfavor*"	Attitude	Product	-
**18 Personal pro-environmental norms**	"*the belief that the individual and other social actors have an obligation to alleviate environmental problems*" (Stern et al., 1999, p. 91)	Norm	Planet, personal practice, without the exclusion of the product and public scope	Ecological citizenship (Seyfang, 2005) Sustainability citizenship (Barry, 2005)
**19 Personal norms to act pro-environmentally**	*"personal feeling of obligation to act pro-environmentally"* (Bouman et al., 2020, p. 4).	Norm	Personal practice, without the exclusion of the product and public scope	-
**20 General pro-environmental intentions**	*"a more general intention to make efforts to protect the environment"* (Lalot et al., 2019, p. 83)	Intention	Personal practice	-
**21 Consciousness for sustainable consumption**	*"an intention to consume in a way that enhances the environmental, social and economic aspects of quality of life"* (Balderjahn et al., 2013, p. 182)	Intention Items: beliefs, values	Product	-
**22 Green purchase intention**	*"the likelihood that a consumer would buy a particular product resulting from his or her environmental needs"* with (Chen and Chang, 2012, p. 507) referring to (Netemeyer et al., 2005) and (Morrison, 1979),	Intention	Product	Purchase intention for environmentally sustainable products (PI) (Kumar et al., 2017)

**23 Ecological behavior**	*"actions which contribute towards environmental preservation and/or conservation."* (Axelrod and Lehman, 1993, p. 153)" as cited by Kaiser et al. (1999b, p. 1).	Behavior	Product, personal practice, planet	Pro-environmental behavior (Bamberg and Möser, 2007)<br>Conservation behavior (Kaiser and Wilson, 2004)<br>Environmentally-friendly behavior (Liobikiene and Juknys, 2016)<br>Environmentally responsible behavior (Thøgersen, 2004)<br>Environmental behavior (Steg and Vlek, 2009)
**24 Environmentalism**	*"defined behaviourally as the propensity to take actions with proenvironmental intent"* (Stern, 2000, p. 411).	Behavior	Product, personal practice, planet	Environmentally significant consumer behavior (Stern, 2000) (Gatersleben et al., 2002)<br>Non-activist support for the environmental movement (Stern et al., 1999)
**25 Sustainable consumption behavior**	*"individual* ***acts*** *of satisfying needs in different areas of life by acquiring, using and disposing goods and services that do not compromise the ecological and socio-economic conditions of all people (currently living or in the future) to satisfy their own needs"* (Geiger et al., 2018, p.5).	Behavior	Product, personal practice	Sustainable consumer behavior (Trudel, 2018)
**26 Environmentally responsible consumption**	*"any consumption-related behaviour, namely, acquisition, use, and disposal, undertaken in a manner such that it reduces the negative impact of consumption on the environment"* (Gupta and Agrawal, 2018, p. 525)	Behavior	Product, personal practice	-

**27 Sustainable lifestyles**	*"patterns of action and consumption, used by people to affiliate and differentiate themselves from others, which: meet basic needs, provide a better quality of life, minimize the use of natural resources and emissions of waste and pollutants over the lifestyle, and do not jeopardize the needs of future generations"* (CSD, 2004, p. 48)	Behavior	Product, personal practice	Ecological lifestyles (Arnold et al., 2018)<br>Green lifestyles (Lorenzen, 2012)<br>Pro-environmental behaviors (Gatersleben et al., 2010)<br>Sustainable development lifestyle (Starcic et al., 2018)
**28 Ecologically conscious consumer behavior**	*"the ecologically conscious consumer can be defined as one who purchases (avoids) products and services which he or she perceives to have a positive (negative) impact on the environment"* (Roberts and Bacon, 1997, p. 84)	Behavior	Product<br>Items: product, personal practice	Green purchase behavior (Chan, 2001)<br>Responsible consumer behavior (Buerke et al., 2017)<br>Green product consumer choice behavior (Lin and Huang, 2012)
**29 Environmentally-motivated consumption reduction**	"*the extent to which consumers lower their consumption in certain domains with the explicit intent to protect the environment*" (Lasarov et al., 2019, p. 282)	Behavior	Product, personal practice	-
**30 Environmentally oriented anti-consumption**	"*acts directed against any form of consumption, with the specific aim of protecting the environment"* (García-de-Frutos et al., 2018, p. 413).	Behavior	Product, personal practice	-
**31Environmental actions**	"*intentional and conscious civic behaviors that are focused on systemic causes of environmental problems and the promotion of environmental sustainability through collective efforts"* (Alisat and Riemer, 2015, p. 14)	Behavior	Public	-

**32 Environmental citizenship**	*"the engagement in political activities aimed at supporting environmental causes"* (Takahashi et al., 2017, p. 114).	Behavior	Public	Green citizenship (Dean, 2001) Environmental citizenship behavior (Song et al., 2019) Pro-environmental activist behavior (Schmitt et al., 2019) Environmental activism (Lee, Lee, Ma, & Cheung, 2019; Steg et al., 2011)
**33a Environmental concern**	*"the affect (i.e., worry) associated with beliefs about environmental problems"* (Schultz et al., 2004, p. 41)	Attitude, belief	Product, personal practice	-
**33b Environmental concern**	*"the degree to which people are aware of problems regarding the environment and support efforts to solve them and/or indicates a willingness to contribute personally to their solution"* (Dunlap and Jones, 2002, p. 485)	Beliefs, behaviors, attitudes	Planet, personal practice Items: planet, public, personal practice, product	-
**34 Environmental consciousness**	*"multi-dimensional construct, consisting of cognitive, attitudinal and behavioural components*" (Schlegelmilch et al., 1996, p. 41)	Knowledge, attitude behavior	Planet (knowledge), Public and planet (attitudes and norms) , product, personal practice, public (behavior)	Ecological concern (Bohlen et al., 1993)

Guiding framework for the choice of the construct of interest

Figure S1 Guiding framework for the selection of constructs (Part 1: concept types: values, identities, knowledge, beliefs, attitudes and norms)

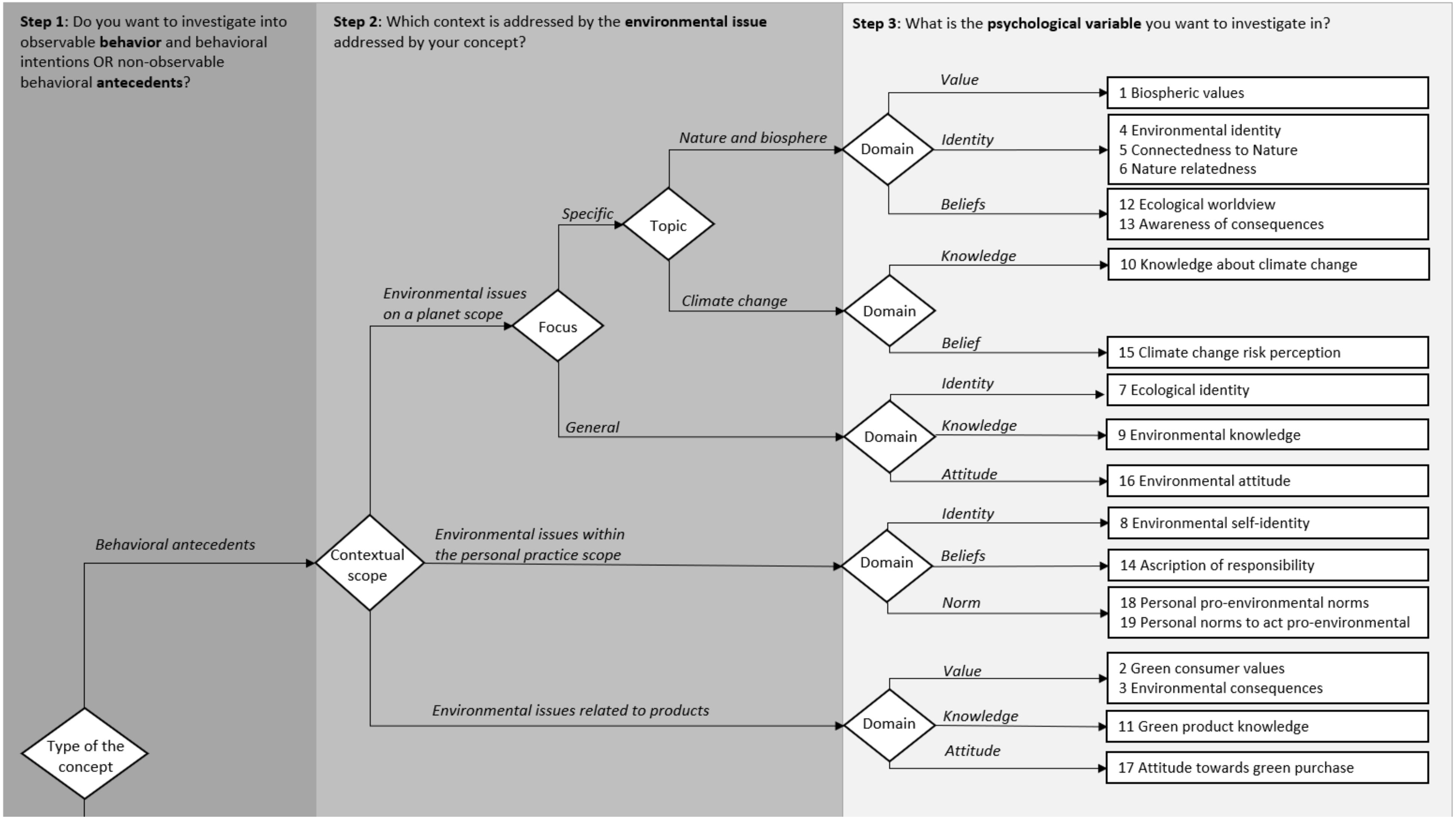

Figure S2 Guiding framework for the selection of constructs (Part 2: concept types: intentions, behaviors, and concepts with multiple concept types)

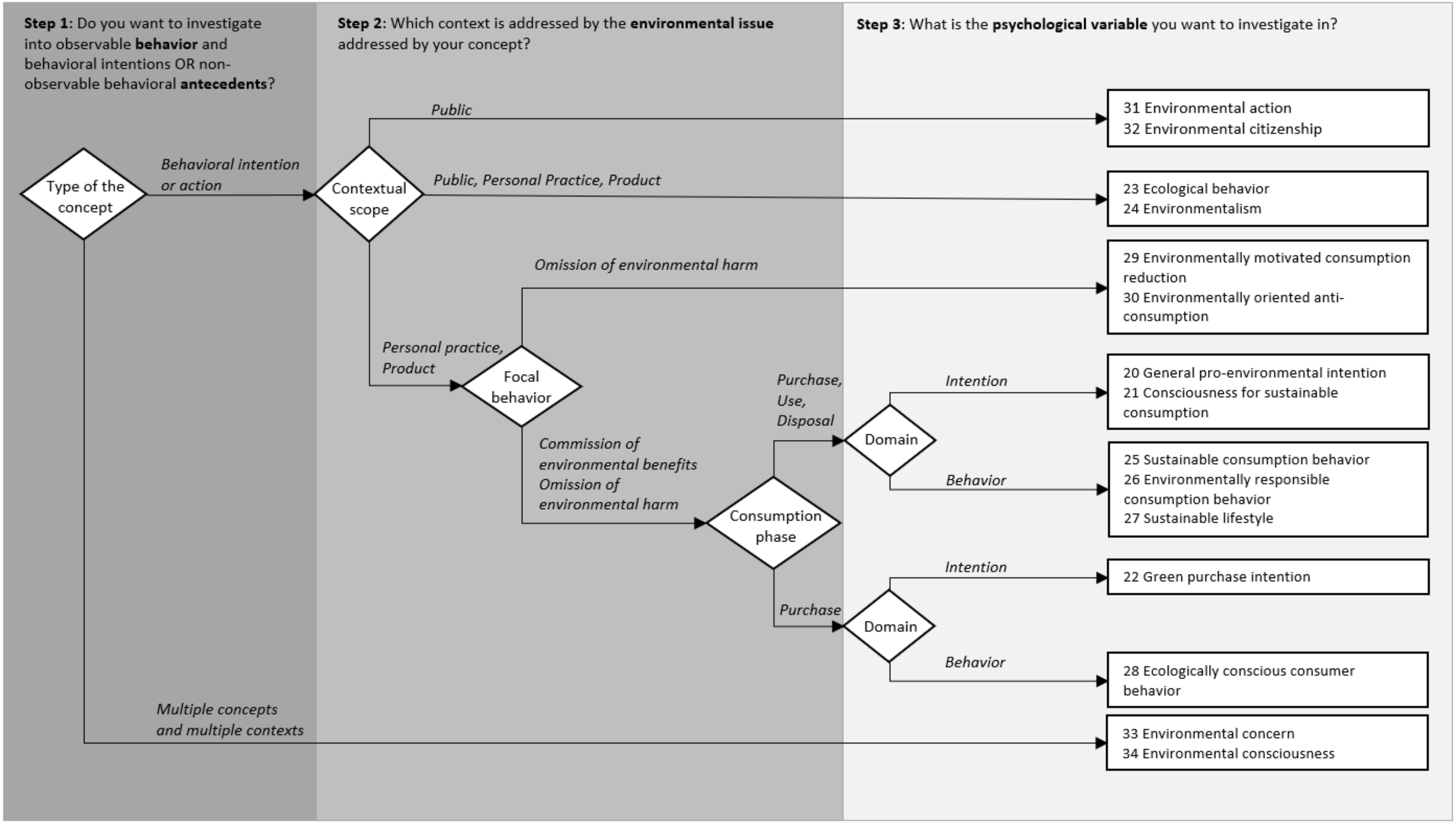

List of scales

This list of tables is provided to facilitate the overview on a total of 76 scales and measures identified in the course of the review on constructs describing and assessing individual-level environmental sustainability. Due to the number of scales and relevant revisions, the list does not include each revision of a scale, but only the latest version or most frequently used version. The scales are listed according to the numbering of the relevant construct in the taxonomy. Scales that were additionally identified in the review but not mentioned in the main part of this paper are ordered chronologically.

Table S2

List of scales identified for each focal construct

Construct	Scales
**1 Biospheric values**	• Schwartz value survey - Biospheric values (Schwartz, 1992)<br>• Environmental portrait value survey – biospheric values (Bouman et al., 2018)
**2 Green consumer value**	• GREEN scale (Haws et al., 2014)<br>• Sustainability focused value orientation (Buerke et al., 2017)
**3 Environmental consequences**	• Environmental consequences measure (Ramayah et al., 2010)
**4 Environmental identity**	• Environmental identity scale (EID) (Clayton, 2003)
**5 Connectedness to nature**	• Emotional affinity towards nature scale (Müller and Kals, 2008)<br>• Connectedness to nature scale (CNS) (Mayer and Frantz, 2004)<br>• Inclusion for nature in self measure (INS) (Schultz, 2002)<br>• Disposition to connect with nature scale (Brügger et al., 2011)
**6 Nature relatedness**	• Nature relatedness scale (NR Scale) (Nisbet et al., 2009)<br>• Nature relatedness scale (NR6 Scale) (Nisbet and Zelenski, 2013)
**7 Ecological Identity**	• Ecological identity scale (Walton and Jones, 2017)
**8 Environmental self-identity**	• Pro-environmental self-identity measure (Whitmarsh and O’Neill, 2010) adapted from (Cook et al., 2002; Sparks and Shepherd, 1992), also used to assess environmentalist identity in an adapted version (Kashima et al., 2014)

	• Environmental self-identity measure (Van Der Werff et al., 2013)
**9 Environmental knowledge**	• Environmental knowledge scale (Lo and Fryxell, 2003)<br>• Environmental knowledge scale (Frick et al., 2004)<br>• Environmental knowledge test (Geiger et al., 2019)
**10 Knowledge about climate change**	• Climate-related knowledge scale<br>• Knowledge about climate change (van der Linden, 2015)
**11 Green product knowledge**	• Environmental knowledge measure (Kumar et al., 2017b) adapted items from the research of (Ramayah et al., 2010)<br>• Green product knowledge measure (Wang et al., 2019) used items from the research of (Liobikiene et al., 2016)<br>• Green product information measure (Ritter et al., 2015)
**12 Ecological worldview**	• New ecological paradigm (NEP)revised scale (Dunlap et al., 2000)
**13 Awareness of consequences**	• General awareness of consequences (GAC) scale (Stern et al., 1995)<br>• Societal consumer instrumentally awareness (Buerke et al., 2017)
**14 Ascription of responsibility**	• Ascription of responsibility (AR) scale (Steg and Groot, 2010)<br>• Perceived consumer effectiveness (Kim and Choi, 2005; Lee et al., 2014)<br>• Ascription to responsibility to self-scale Adapted from previous research within the marketing literature (Peloza et al., 2013) by (Wu and Yang, 2018)
**15 Climate change risk perception**	• Risk perception index items (van der Linden, 2015)<br>• Concern about climate change scale (Tobler et al., 2012)<br>• Climate concern measure (Alcock et al., 2017)<br>• Climate change belief measure (Brick, Sherman, & Kim, 2017)
**16 Environmental attitude**	• Environmental attitude and knowledge scale (EAKS) (Maloney et al., 1975)<br>• Environmental attitude related scales (Kaiser et al., 1999a)<br>• Environmental attitudes inventory (EIA) (Milfont and Duckitt, 2010)<br>• Pro environmental attitude (Lavelle et al., 2015)

**17 Attitude towards green purchase**	• Attitude towards green purchase (Chan, 2001)
**18 Personal pro-environmental norms**	• Personal pro-environmental norms measure (P. C. Stern et al., 1999)
**19 Personal norms to act pro-environmentally**	• Personal norms to act pro-environmentally measure (Steg et al., 2011)<br>• Personal norms measure I (Steg and Groot, 2010)<br>• Personal norms measure II (Van der Werff et al., 2013)<br>• Personal norms measure III (Verplanken and Roy, 2016)
**20 General pro-environmental intentions**	• General pro-environmental intention scale (Lalot et al., 2019)
**21 Consciousness for sustainable consumption**	• Consciousness for sustainable consumption (CSC) scale (Balderjahn et al., 2013)
**22 Green purchase intention**	• Green purchase intention measure (Chen and Chang, 2012)<br>• Purchase intention for environmentally sustainable products measure (Kumar et al., 2017)
**23 Ecological behavior**	• General ecological behavior scale (GEB) (Kaiser, 1998), most recent version of GEB applied by (Arnold et al., 2018)<br>• General responsible environmental behavior measure (Cottrell, 2003)<br>• Environmentally relevant behaviors measure (Thøgersen, 2004)<br>• Environmental behaviors measure (Kilbourne and Pickett, 2008)<br>• Pro-environmental behavior scale based on (DEFRA, 2008) adapted by (Whitmarsh and O'Neill, 2010)<br>• Pro-environmental behavior scale (Markle, 2013)<br>• Three types of pro-environmental behavior measure (Lee et al., 2014)<br>• Recurring pro-environmental behavior scale (REBS) (Brick, Sherman, & Kim, 2017)
**24 Environmentalism**	• Environmentalism scale (Stern, 2000)<br>• Types of pro-environmental behaviors measure (Larson et al., 2015)
**25 Sustainable consumption behavior**	• Sustainable consumption behavior measure I (Wang et al., 2014)<br>• Sustainable consumption behavior measure II (Watkins et al., 2016)

	• Young consumers' sustainable consumption behaviors (YCSCB) scale in the areas of food and clothing (Fischer et al., 2017)
**26 Environmentally responsible consumption**	• Environmentally responsible consumption scale (Gupta and Agrawal, 2018)
**27 Sustainable lifestyles**	• Sustainable lifestyles measure (Barr and Gilg, 2006)<br>• Pro-environmental behaviors measure (Gatersleben et al., 2010)<br>• Sustainable development lifestyle measure (Starcic et al., 2018)
**28 Ecologically conscious consumer behavior**	• Ecologically conscious consumer behavior (ECCB) scale (Roberts and Bacon, 1997)<br>• Green product consumer choice scale (Lin and Huang, 2012)<br>• Responsible consumer behaviour scale (Buerke et al., 2017)
**29 Environmentally-motivated consumption reduction**	• Environmentally motivated consumption reduction scale (Egea and de Frutos, 2013)
**30 Environmentally oriented anti-consumption**	• EOA has not yet been operationalized. Ortega Egea & García de Frutos (2020) use behavioral measures from the five Eurobarometer studies on climate change (i.e., Eurobarometers 77.1, 75.4, 80.3, 83.4, and 87.1; see GESIS - Leibniz Institute for the Social Sciences, 2020) to approximate EOA).
**31Environmental actions**	• Environmental action scale (Alisat and Riemer, 2015)
**32 Environmental citizenship**	• Environmental citizenship measure (Takahashi et al., 2017)
**33 Environmental concern**	• Environmental concern measure I (Weigel and Weigel, 1978)<br>• Environmental concern measure II (Schultz, 2001)<br>• Environmental concern measure III (Bamberg, 2003) based on (Preisendörfer, 1996)<br>• Environmental concern measure IV (Kilbourne and Pickett, 2008)<br>• Environmental concern measure V (Mohd Suki, 2016) based on (Tarrant and Cordell, 1997)
**34 Environmental consciousness**	• Environmental consciousness scale (Diamantopoulos et al., 2003)<br>• Ecological consciousness measure (Tilikidou et al., 2002)

Guidance for researchers dedicated to enhancing conceptual rigor and measurement quality in the field – Examples for relevant constructs

**Lack of discriminant validity among concepts**
Our review revealed a total of 38 synonymous concepts (Table S1) and 16 constructs (Construct number: 5-8, 18, 19, 25-32 in Table S1) with partially overlapping domains and facets. In particular, identity, normative, and behavioral concepts are subject to the latter. This often results from the parallel introduction of similar conceptual ideas in different literature streams and render it difficult to grasp the current knowledge across the literature. This can followingly yield a scattered knowledge base (MacKenzie, 2003; Mochon and Schwartz, 2020), which is disadvantageous for advancing knowledge appropriately.
**Conceptually distinct concepts carrying identical names**
Our review revealed concepts carrying identical names but capturing distinct conceptual ideas (i.e., connectedness to nature, environmental attitudes, and environmental concern). For these concepts the literature offers multiple conceptualizations that considerably differ in their respective understanding of the focal concept. In some cases, such conceptual divides are explicitly discussed in the literature and justified by different conceptual perspectives among authors (for example, conceptualizing connectedness to nature as either an identity (Mayer and Frantz, 2004) or an attitude (Brügger et al., 2011). In other cases, such differences are neither explicitly addressed or theoretically delineated (for example, conceptualizing environmental concern as an exclusively attitudinal construct (Schultz et al., 2004) or as a multi-conceptual construct (Dunlap and Jones, 2002)), but rather seem to result from neglect to adequately account for related concepts when introducing additional concepts. While explicit disagreement on the conceptual core of concepts is legitimate, unintentional reasons for inconsistent conceptualizations should be avoided. In both cases, researchers using these concepts in their research should aim to have a clear understanding of the concept type of a construct at hand, as this is the basis for interpreting substantive research findings (MacKenzie, 2003).
**Inconsistent operationalization**
Our review identified a total of 10 constructs (Construct number: 2, 4-8, 9, 11, 21, 28, 29 in Table S1) subject to a considerable divide between their conceptual domain on the one hand and their operationalization on the other. In other words, the measurement items do not (fully) correspond to the concept core defined in the conceptual definition. Instead, the items tap other or additional facets not reflected in the underlying concept description (illustrative examples include green consumption values (Haws et al., 2014) and ECCB (Roberts and Bacon, 1997)). Such divides are critical because measures that fail to essentially cover the conceptual domains are likely to cause misinterpretation of substantive findings (DeVellis, 2016; Mochon and Schwartz, 2020).

References

Alcock, I., White, M.P., Taylor, T., Coldwell, D.F., Gribble, M.O., Evans, K.L., Corner, A., Vardoulakis, S., Fleming, L.E., 2017. 'Green' on the ground but not in the air: Pro-environmental attitudes are related to household behaviours but not discretionary air travel. Global Environmental Change 42, 136–147. https://doi.org/10.1016/j.gloenvcha.2016.11.005

Alisat, S., Riemer, M., 2015. The environmental action scale: Development and psychometric evaluation. Journal of Environmental Psychology 43, 13–23. https://doi.org/10.1016/j.jenvp.2015.05.006

Arnold, O., Kibbe, A., Hartig, T., Kaiser, F.G., 2018. Capturing the Environmental Impact of Individual Lifestyles: Evidence of the Criterion Validity of the General Ecological Behavior Scale. Environment and Behavior 50, 350–372. https://doi.org/10.1177/0013916517701796

Axelrod, L.J., Lehman, D.R., 1993. Responding to environmental concerns: What factors guide individual action? Journal of Environmental Psychology 13, 149–159. https://doi.org/10.1016/S0272-4944(05)80147-1

Balderjahn, I., Buerke, A., Kirchgeorg, M., Peyer, M., Seegebarth, B., Wiedmann, K.-P., 2013. Consciousness for sustainable consumption: scale development and new insights in the economic dimension of consumers' sustainability. AMS Review 3, 181–192. https://doi.org/10.1007/s13162-013-0057-6

Bamberg, S., 2003. How does environmental concern influence specific environmentally related behaviors? A new answer to an old question. Journal of Environmental Psychology 23, 21–32. https://doi.org/10.1016/S0272-4944(02)00078-6

Bamberg, S., Möser, G., 2007. Twenty years after Hines, Hungerford, and Tomera: A new meta-analysis of psycho-social determinants of pro-environmental behaviour. Journal of Environmental Psychology 27, 14–25. https://doi.org/10.1016/j.jenvp.2006.12.002

Barr, S., Gilg, A., 2006. Sustainable lifestyles: Framing environmental action in and around the home. Geoforum 37, 906–920. https://doi.org/10.1016/j.geoforum.2006.05.002

Barry, J., 2005. Resistance Is Fertile: From Environmental to Sustainability Citizenship.

Bohlen, G., Schlegelmilch, B.B., Diamantopoulos, A., 1993. Measuring ecological concern: A multi-construct perspective. Journal of Marketing Management 9, 415–430. https://doi.org/10.1080/0267257X.1993.9964250

Bouman, T., Steg, L., Johnson-Zawadzki, S., 2020. The value of what others value: When perceived biospheric group values influence individuals' pro-environmental engagement. Journal of Environmental Psychology 71, 101470. https://doi.org/10.1016/j.jenvp.2020.101470

Bouman, T., Steg, L., Kiers, H.A.L., 2018. Measuring values in environmental research: A test of an environmental Portrait Value Questionnaire. Frontiers in Psychology 9, 1–15. https://doi.org/10.3389/fpsyg.2018.00564

Brick, C., Sherman, D.K., Kim, H.S., 2017a. "Green to be seen" and "brown to keep down": Visibility moderates the effect of identity on pro-environmental behavior. Journal of Environmental Psychology 51, 226–238. https://doi.org/10.1016/j.jenvp.2017.04.004

Brick, C., Sherman, D.K., Kim, H.S., 2017b. "Green to be seen" and "brown to keep down": Visibility moderates the effect of identity on pro-environmental behavior. Journal of Environmental Psychology 51, 226–238. https://doi.org/10.1016/j.jenvp.2017.04.004

Brügger, A., Kaiser, F.G., Roczen, N., 2011. One for all?: Connectedness to nature, inclusion of nature, environmental identity, and implicit association with nature. European Psychologist. https://doi.org/10.1027/1016-9040/a000032

Buerke, A., Straatmann, T., Lin-Hi, N., Müller, K., 2017. Consumer awareness and sustainability-focused value orientation as motivating factors of responsible consumer behavior. Review of Managerial Science 11, 959–991. https://doi.org/10.1007/s11846-016-0211-2

Chan, R.Y.K., 2001. Determinants of Chinese Consumers ' Green Purchase Behavior. Psychology and Marketing 18, 389–413.

Chen, Y., Chang, C., 2012. Enhance green purchase intentions. Management Decision 50,

502–520. https://doi.org/10.1108/00251741211216250
Clayton, S., 2003. Environmental Identity: A conceptual and an operational definition, in: Clayton, S., Opotow, S. (Eds.), Identity and the Natural Environment. MIT PRess, Cambridge, MA.
Cook, A.J., Kerr, G.N., Moore, K., 2002. Attitudes and intentions towards purchasing GM food. Journal of Economic Psychology 23, 557–572.
Cottrell, S.P., 2003. Influence of sociodemographics and environmental attitudes on general responsible environmental behavior among recreational boaters. Environment and Behavior 35, 347–375. https://doi.org/10.1177/0013916503035003003
CSD, 2004. Every little bit helps…" Overcoming the challenges to researching, promoting and implementing sustainable lifestyles. London.
Dean, H., 2001. Green citizenship. Social Policy and Administration 35, 490–505. https://doi.org/10.1111/1467-9515.t01-1-00249
DEFRA, 2008. A framework for pro-environmental behaviours. London.
Dermody, J., Koenig-Lewis, N., Zhao, A.L., Hanmer-Lloyd, S., 2018. Appraising the influence of pro-environmental self-identity on sustainable consumption buying and curtailment in emerging markets: Evidence from China and Poland. Journal of Business Research 86, 333–343. https://doi.org/10.1016/j.jbusres.2017.09.041
Diamantopoulos, A., Schlegelmilch, B.B., Sinkovics, R.R., Bohlen, G.M., 2003. Can socio-demographics still play a role in profiling green consumers? A review of the evidence and an empirical investigation. Journal of Business Research. https://doi.org/10.1016/S0148-2963(01)00241-7
Dunlap, R., Jones, R., 2002. Environmental Concern: Conceptual and Measurement Issues, in: Dunlap, Michelson (Eds.), Handbook of Environmental Sociology. Greenwood Press, London, pp. 482–542.
Dunlap, R., Van Liere, K., 1978. The "New Environmental Paradigm." The Journal of Environmental Education 9, 10–19. https://doi.org/10.1080/00958964.1978.10801875
Dunlap, R., Van Liere, K.D., Mertig, A.G., Jones, R.E., 2000. New Trends in Measuring Environmental Attitudes: Measuring Endorsement of the New Ecological Paradigm: A Revised NEP Scale. Journal of Social Issues 56, 425–442. https://doi.org/10.1111/0022-4537.00176
Dutcher, D.D., Finley, J.C., Luloff, A.E., Johnson, J.B., 2007. Connectivity With Nature as a Measure of Environmental Values. Environment and Behavior 39, 474–493. https://doi.org/10.1177/0013916506298794
Eagly, A.H., Chaiken, S., 1993. The psychology of attitudes.
Egea, J.M.O., de Frutos, N.G., 2013. Toward Consumption Reduction: An Environmentally Motivated Perspective. Psychology & Marketing 30, 660–675. https://doi.org/10.1002/mar.20636
Fischer, D., Böhme, T., Geiger, S.M., 2017. Measuring young consumers' sustainable consumption behavior: development and validation of the YCSCB scale. Young Consumers 18. https://doi.org/10.1108/YC-03-2017-00671
Frick, J., Kaiser, F.G., Wilson, M., 2004. Environmental knowledge and conservation behavior: Exploring prevalence and structure in a representative sample. Personality and Individual Differences 37, 1597–1613. https://doi.org/10.1016/j.paid.2004.02.015
García-de-Frutos, N., Ortega-Egea, J.M., Martínez-del-Río, J., 2018. Anti-consumption for Environmental Sustainability: Conceptualization, Review, and Multilevel Research Directions. Journal of Business Ethics 148, 411–435. https://doi.org/10.1007/s10551-016-3023-z
Gatersleben, B., Steg, L., Vlek, C., 2002. Measurement and determinants of environmentally significant consumer behavior. Environment and Behavior 34, 335–362. https://doi.org/10.1177/0013916502034003004
Gatersleben, B., White, E., Abrahamse, W., Jackson, T., Uzzell, D., 2010. Values and sustainable lifestyles. Architectural Science Review 53, 37–50. https://doi.org/10.3763/asre.2009.0101
Geiger, S.M., Fischer, D., Schrader, U., 2018. Measuring What Matters in Sustainable Consumption: An Integrative Framework for the Selection of Relevant Behaviors

Introduction: The Necessity and Difficulty of Measuring the Sustainability of Consumption Behavior. Sustainable Development 18–33. https://doi.org/10.1002/sd.1688

Geiger, S.M., Geiger, M., Wilhelm, O., 2019. Environment-Specific vs. General Knowledge and Their Role in Pro-environmental Behavior. Frontiers in Psychology 10, 1–12. https://doi.org/10.3389/fpsyg.2019.00718

GESIS - Leibniz Institute for the Social Sciences, 2020. Eurobarometer Data Service [WWW Document]. Standard & Special EB Study Profiles. URL https://www.gesis.org/en/eurobarometer-data-service/survey-series/standard-special-eb/study-overview

Gupta, S., Agrawal, R., 2018. Environmentally responsible consumption: Construct definition, scale development, and validation. Corporate Social Responsibility and Environmental Management 25, 523–536. https://doi.org/10.1002/csr.1476

Haws, K.L., Winterich, K.P., Naylor, R.W., 2014. Seeing the world through GREEN-tinted glasses: Green consumption values and responses to environmentally friendly products. Journal of Consumer Psychology 24, 336–354. https://doi.org/10.1016/j.jcps.2013.11.002

Kaiser, F.G., 1998. A general measure of ecological behavior. Journal of Applied Social Psychology 28, 395–422. https://doi.org/10.1111/j.1559-1816.1998.tb01712.x

Kaiser, F.G., Ranney, M., Hartig, T., Bowler, P.A., 1999a. Ecological Behavior, Environmental Attitude, and Feelings of Responsibility for the Environment. European Psychologist 4, 59–74.

Kaiser, F.G., Wilson, M., 2004. Goal-directed conservation behavior: The specific composition of a general performance. Personality and Individual Differences 36, 1531–1544. https://doi.org/10.1016/j.paid.2003.06.003

Kaiser, F.G., Wölfing, S., Fuhrer, U., 1999b. Environmental Attitude and Ecolgocial Behaviour. Journal of Environmental Psychology 19, 1–19.

Kals, E., Schumacher, D., Montada, L., 1999. Emotional affinity toward nature as a motivational basis to protect nature. Environment and Behavior 31, 178–202. https://doi.org/10.1177/00139169921972056

Kashima, Y., Paladino, A., Margetts, E.A., 2014. Environmentalist identity and environmental striving. Journal of Environmental Psychology 38, 64–75. https://doi.org/10.1016/j.jenvp.2013.12.014

Kilbourne, W., Pickett, G., 2008. How materialism affects environmental beliefs, concern, and environmentally responsible behavior. Journal of Business Research 61, 885–893. https://doi.org/10.1016/j.jbusres.2007.09.016

Kim, Y., Choi, S.M., 2005. Antecedents of green purchase behavior: An examination of collectivism, environmental concern, and PCE. Advances in consumer research 32, 592–599.

Kumar, B., Manrai, A.K., Manrai, L.A., 2017a. Purchasing behaviour for environmentally sustainable products: A conceptual framework and empirical study. Journal of Retailing and Consumer Services 34, 1–9. https://doi.org/10.1016/j.jretconser.2016.09.004

Kumar, B., Manrai, A.K., Manrai, L.A., 2017b. Purchasing behaviour for environmentally sustainable products: A conceptual framework and empirical study. Journal of Retailing and Consumer Services 34, 1–9. https://doi.org/10.1016/j.jretconser.2016.09.004

Lalot, F., Quiamzade, A., Falomir-Pichastor, J.M., Gollwitzer, P.M., 2019. When does self-identity predict intention to act green? A self-completion account relying on past behaviour and majority-minority support for pro-environmental values. Journal of Environmental Psychology 61, 79–92. https://doi.org/10.1016/j.jenvp.2019.01.002

Larson, L.R., Stedman, R.C., Cooper, C.B., Decker, D.J., 2015. Understanding the multi-dimensional structure of pro-environmental behavior. Journal of Environmental Psychology 43, 112–124. https://doi.org/10.1016/j.jenvp.2015.06.004

Lasarov, W., Mai, R., García de Frutos, N., Egea, J.M.O., Hoffmann, S., 2019. Counter-arguing as barriers to environmentally motivated consumption reduction: A multi-country study. International Journal of Research in Marketing 36, 281–305. https://doi.org/10.1016/j.ijresmar.2018.11.005

Lavelle, M.J., Rau, H., Fahy, F., 2015. Different shades of green? Unpacking habitual and occasional pro-environmental behavior. Global Environmental Change 35, 368–378.

https://doi.org/10.1016/j.gloenvcha.2015.09.021
Lee, K.M.Y., Lee, J.C.K., Ma, A.T.H., Cheung, L.T.O., 2019. Does human rights awareness spur environmental activism? Hong Kong's 'country park' controversy. Land Use Policy 87, 104033. https://doi.org/10.1016/j.landusepol.2019.104033
Lee, Y. ki, Kim, S., Kim, M. seong, Choi, J. gu, 2014. Antecedents and interrelationships of three types of pro-environmental behavior. Journal of Business Research 67, 2097–2105. https://doi.org/10.1016/j.jbusres.2014.04.018
Leiserowitz, A., Smith, N., Project, Y., Gaddis, J., Fernandez, L., Read, D., 2010. Americans' Knowledge of Climate Change, Yale Project on Climate Change Communication. New Haven.
Lin, P.-C., Huang, Y.-H., 2012. The influence factors on choice behavior regarding green products based on the theory of consumption values. Journal of Cleaner Production 22, 11–18. https://doi.org/10.1016/j.jclepro.2011.10.002
Liobikiene, G., Juknys, R., 2016. The role of values, environmental risk perception, awareness of consequences, and willingness to assume responsibility for environmentally-friendly behaviour: The Lithuanian case. Journal of Cleaner Production 112, 3413–3422. https://doi.org/10.1016/j.jclepro.2015.10.049
Liobikiene, G., Mandravickaite, J., Bernatoniene, J., 2016. Theory of planned behavior approach to understand the green purchasing behavior in the EU: A cross-cultural study. Ecological Economics 125, 38–46. https://doi.org/10.1016/j.ecolecon.2016.02.008
Lo, C.W.H., Fryxell, G.E., 2003. The Influence of Environmental Knowledge and Values on Managerial Behaviours on Behalf of the Environment: An Empirical Examination of Managers in China. Journal of Business Ethics 46, 45–69.
Lorenzen, J.A., 2012. Going Green: The Process of Lifestyle Change1. Sociological Forum 27, 94–116. https://doi.org/10.1111/j.1573-7861.2011.01303.x
Maloney, M.P., Ward, M.P., Braucht, G.N., 1975. A revised scale for the measurement of ecological attitudes and knowledge. American Psychologist 30, 787–790. https://doi.org/10.1037/h0084394
Markle, G.L., 2013. Pro-Environmental Behavior: Does It Matter How It's Measured? Development and Validation of the Pro-Environmental Behavior Scale (PEBS). Human Ecology 41, 905–914. https://doi.org/10.1007/s10745-013-9614-8
Mayer, F.S., Frantz, C.M.P., 2004. The connectedness to nature scale: A measure of individuals' feeling in community with nature. Journal of Environmental Psychology 24, 503–515. https://doi.org/10.1016/j.jenvp.2004.10.001
Milfont, T., 2007. Psychology of Environmental Attitudes: A cross-cultural study of their content and structure. ResearchSpace@Auckland 1994, 318.
Milfont, T., Duckitt, J., 2010. The environmental attitudes inventory: A valid and reliable measure to assess the structure of environmental attitudes. Journal of Environmental Psychology 30, 80–94. https://doi.org/10.1016/j.jenvp.2009.09.001
Mohd Suki, N., 2016. Consumer environmental concern and green product purchase in Malaysia: structural effects of consumption values. Journal of Cleaner Production 132, 204–214. https://doi.org/10.1016/j.jclepro.2015.09.087
Morrison, D.G., 1979. Purchase Intentions and Purchase Behavior. Journal of Marketing 43, 65. https://doi.org/10.2307/1250742
Müller, M.M., Kals, E., 2008. Adolescents ' Emotional Affinity toward Nature : A Cross-Societal Study 59–69.
Netemeyer, R.G., Maxham, J.G., Pullig, C., 2005. Conflicts in the Work–Family Interface: Links to Job Stress, Customer Service Employee Performance, and Customer Purchase Intent. Journal of Marketing 69, 130–143. https://doi.org/10.1509/jmkg.69.2.130.60758
Nisbet, E.K., Zelenski, J.M., 2013. The NR-6: A new brief measure of nature relatedness. Frontiers in Psychology 4, 1–11. https://doi.org/10.3389/fpsyg.2013.00813
Nisbet, E.K., Zelenski, J.M., Murphy, S.A., 2009. The Nature Relatedness Scale. Environment and Behavior 41, 715–740. https://doi.org/10.1177/0013916508318748
Ortega Egea, J.M., García de Frutos, N., 2020. Behavioral prediction of environmentally oriented anticonsumption and consumption: A multilevel study of five Eurobarometer surveys. Psychology & Marketing 37, 308–325. https://doi.org/10.1002/mar.21302

Peloza, J., White, K., Shang, J., 2013. Good and Guilt-Free: The Role of Self-Accountability in Influencing Preferences for Products with Ethical Attributes. Journal of Marketing 77, 104–119. https://doi.org/10.1509/jm.11.0454

Preisendörfer, P., 1996. Umweltbewusstsein in Deutschland. Ergebnisse einer repräsentativen Bevölkerungsumfrage. Bonn.

Ramayah, T., Lee, J.W.C., Mohamad, O., 2010. Green product purchase intention: Some insights from a developing country. Resources, Conservation and Recycling 54, 1419–1427. https://doi.org/10.1016/j.resconrec.2010.06.007

Ritter, Á.M., Borchardt, M., Vaccaro, G.L.R., Pereira, G.M., Almeida, F., 2015. Motivations for promoting the consumption of green products in an emerging country: exploring attitudes of Brazilian consumers. Journal of Cleaner Production 106, 507–520. https://doi.org/10.1016/j.jclepro.2014.11.066

Roberts, J.A., Bacon, D.R., 1997. Exploring the Subtle Relationships between Environmental Concern and Ecologically Conscious Consumer Behavior. Journal of Business Research.

Schlegelmilch, B.B., Bohlen, G.M., Diamantopoulos, A., 1996. The link between green purchasing decisions and measures of environmental consciousness. European Journal of Marketing 30, 35–55. https://doi.org/10.1108/03090569610118740

Schmitt, M.T., Mackay, C.M.L., Droogendyk, L.M., Payne, D., 2019. What predicts environmental activism? The roles of identification with nature and politicized environmental identity. Journal of Environmental Psychology 61, 20–29. https://doi.org/10.1016/j.jenvp.2018.11.003

Schultz, P.W., 2001. The structure of environmental concern: Concern for self, other people, and the biosphere. Journal of Environmental Psychology 21, 327–339. https://doi.org/10.1006/jevp.2001.0227

Schultz, P.W., Shriver, C., Tabanico, J.J., Khazian, A.M., 2004. Implicit connections with nature. Journal of Environmental Psychology 24, 31–42. https://doi.org/10.1016/S0272-4944(03)00022-7

Schultz, W.P., 2002. Inclusion with Nature: The psychology of human-nature relations, in: Schmuck, P., Schultz, W.P. (Eds.), Psychology of Sustainable Development. Kluwer Academic Publishers, Boston, MA. https://doi.org/10.1007/978-1-4615-0995-0

Schwartz, S., 1992. Universals in the content and structure of values: theoretical tests in 20 countries. Advances in Experimental Social Psychology 25, 1–65.

Seyfang, G., 2005. Shopping for sustainability: Can sustainable consumption promote ecological citizenship? Environmental Politics 14, 290–306. https://doi.org/10.1080/09644010500055209

Song, Z., Daryanto, A., Soopramanien, D., 2019. Place attachment, trust and mobility: Three-way interaction effect on urban residents' environmental citizenship behaviour. Journal of Business Research 105, 168–177. https://doi.org/10.1016/j.jbusres.2019.08.001

Sparks, P., Shepherd, R., 1992. Self-Identity and the Theory of Planned Behavior: Assesing the Role of Identification with "Green Consumerism." Social Psychology Quarterly 55, 388. https://doi.org/10.2307/2786955

Starcic, A.I., Terlevic, M., Lin, L., Lebenicnik, M., 2018. Designing learning for sustainable development: Digital practices as boundary crossers and predictors of sustainable lifestyles. Sustainability (Switzerland) 10. https://doi.org/10.3390/su10062030

Steg, L., De Groot, J.I.M., Dreijerink, L., Abrahamse, W., Siero, F., 2011. General Antecedents of Personal Norms, Policy Acceptability, and Intentions: The Role of Values, Worldviews, and Environmental Concern. Society & Natural Resources 24, 349–367. https://doi.org/10.1080/08941920903214116

Steg, L., Groot, J., 2010. Explaining prosocial intentions: Testing causal relationships in the norm activation model. British Journal of Social Psychology 49, 725–743. https://doi.org/10.1348/014466609X477745

Steg, L., Vlek, C., 2009. Encouraging pro-environmental behaviour: An integrative review and research agenda. Journal of Environmental Psychology 29, 309–317. https://doi.org/10.1016/j.jenvp.2008.10.004

Stern, P., Dietz, T., Abel, T., Guagnano, G.A., Kalof, L., 1999. A value-belief-norm theory of support for social movements: The case of environmentalism. Human Ecology Review 6,

81–97.
Stern, P.C., 2000. Toward a Coherent Theory of Environmentally Significant Behavior. Journal of Social Issues 56, 407–424.
Stern, P.C., Dietz, T., Abel, T., Guagnano, G.A., Kalof, L., 1999. A value-belief-norm theory of support for social movements: The case of environmentalism. Human Ecology Review 6, 81–97.
Stern, P.C., Dietz, T., Kalof, L., 1993. Value Orientations, Gender, and Environmental Concern. Environment and Behavior 25, 322–348. https://doi.org/10.1177/0013916593255002
Stern, P.C., Kalof, L., Dietz, T., Guagnano, G.A., 1995. Values, Beliefs, and Proenvironmental Action: Attitude Formation Toward Emergent Attitude Objects. Journal of Applied Social Psychology 25, 1611–1636. https://doi.org/10.1111/j.1559-1816.1995.tb02636.x
Takahashi, B., Tandoc, E.C., Duan, R., Van Witsen, A., 2017. Revisiting Environmental Citizenship: The Role of Information Capital and Media Use. Environment and Behavior 49, 111–135. https://doi.org/10.1177/0013916515620892
Tarrant, M.A., Cordell, H.K., 1997. The Effect of Respondent Characteristics on General Environmental Attitude-Behavior Correspondence. Environment and Behavior 29, 618–637. https://doi.org/10.1177/0013916597295002
Thøgersen, J., 2004. A cognitive dissonance interpretation of consistencies and inconsistencies in environmentally responsible behavior. Journal of Environmental Psychology 24, 93–103. https://doi.org/10.1016/S0272-4944(03)00039-2
Tilikidou, I., Adamson, I., Sarmaniotis, C., 2002. The measurement instrument of ecologically-conscious consumer behaviour, in: Intergovernmental Panel on Climate Change (Ed.), Climate Change 2013 - The Physical Science Basis. Cambridge University Press, Cambridge, pp. 1–30. https://doi.org/10.1017/CBO9781107415324.004
Tobler, C., Visschers, V.H.M., Siegrist, M., 2012. Consumers' knowledge about climate change. Climatic Change 114, 189–209. https://doi.org/10.1007/s10584-011-0393-1
Trudel, R., 2018. Sustainable consumer behavior. Consumer Psychology Review 85–96. https://doi.org/10.1002/arcp.1045
van der Linden, S., 2015. The social-psychological determinants of climate change risk perceptions: Towards a comprehensive model. Journal of Environmental Psychology 41, 112–124. https://doi.org/10.1016/j.jenvp.2014.11.012
Van der Werff, E., Steg, L., Keizer, K., 2013. It is a moral issue: The relationship between environmental self-identity, obligation-based intrinsic motivation and pro-environmental behaviour. Global Environmental Change 23, 1258–1265. https://doi.org/10.1016/j.gloenvcha.2013.07.018
Van Der Werff, E., Steg, L., Keizer, K., 2013. The value of environmental self-identity: The relationship between biospheric values, environmental self-identity and environmental preferences, intentions and behaviour. Journal of Environmental Psychology 34, 55–63. https://doi.org/10.1016/j.jenvp.2012.12.006
Verplanken, B., Roy, D., 2016. Empowering interventions to promote sustainable lifestyles: Testing the habit discontinuity hypothesis in a field experiment. Journal of Environmental Psychology 45, 127–134. https://doi.org/10.1016/j.jenvp.2015.11.008
Walton, T.N., Jones, R.E., 2017. Ecological Identity: The Development and Assessment of a Measurement Scale. Environment and Behavior 50, 1–33. https://doi.org/10.1177/0013916517710310
Wang, H., Ma, B., Bai, R., 2019. How Does Green Product Knowledge Effectively Promote Green Purchase Intention? Sustainability 11, 1193. https://doi.org/10.3390/su11041193
Wang, P., Liu, Q., Qi, Y., 2014. Factors influencing sustainable consumption behaviors: a survey of the rural residents in China. Journal of Cleaner Production 63, 152–165. https://doi.org/10.1016/j.jclepro.2013.05.007
Watkins, L., Aitken, R., Mather, D., 2016. Conscientious consumers: a relationship between moral foundations, political orientation and sustainable consumption. Journal of Cleaner Production 134, 137–146. https://doi.org/10.1016/j.jclepro.2015.06.009
Weigel, R., Weigel, J., 1978. Environmental Concern. Environment and Behavior 10, 3–15. https://doi.org/10.1177/0013916578101001

Whitmarsh, L., O'Neill, S., 2010. Green identity, green living? The role of pro-environmental self-identity in determining consistency across diverse pro-environmental behaviours. Journal of Environmental Psychology 30, 305–314. https://doi.org/10.1016/j.jenvp.2010.01.003

Wu, B., Yang, Z., 2018. The impact of moral identity on consumers' green consumption tendency: The role of perceived responsibility for environmental damage. Journal of Environmental Psychology 59, 74–84. https://doi.org/10.1016/j.jenvp.2018.08.011

Zhu, J., Hu, S., Wang, J., Zheng, X., 2020. Future orientation promotes climate concern and mitigation. Journal of Cleaner Production 262, 121212. https://doi.org/10.1016/j.jclepro.2020.121212
