# Supplementary material for: Concepts Describing and Assessing Individuals’ Environmental Sustainability: An Integrative Review and Taxonomy
Source: Front Psychol. 2022 Jan 5;12:770470. doi: 10.3389/fpsyg.2021.770470 (PMC8766306; doi:10.3389/fpsyg.2021.770470)
Supplement: Supplementary file 2 [file Table_1.docx]

**Table A1** *Search strategy with list of search terms*

| Search aspect | Keywords |
| --- | --- |
| 1. Ecological sustainability aspect | Environment* OR Ecologic*OR Green OR Proenvironmental OR Pro-environmental OR Resource-efficient OR Sustainab* |
| 1. Concept type | Value* OR Identit* OR Knowledge OR Belief* OR Attitude* OR Norm* OR Intention* OR Behavi*o* OR Lifestyle* |
| 1. Individual-level | Individual OR Consumer OR Personal OR Self- |
| 1. Contextual scope | Purchase OR Household OR Practice OR Action OR Activism OR Civic OR Nature OR Ecosystem OR Climate OR Planet |
| 1. Conceptualization | Concept OR Conceptualization OR Construct OR Operationalization |
| Search strategy  *Data Base* | Example for a search strategy:  TITLE (1&2) AND TITLE-ABS-KEY (1&2&3&4) AND (5) |
|  | *Scopus* Example for search string^1^:  (TITLE-ABS-KEY ( environment* OR ecologic* OR green OR environmental OR pro-environmental OR resource-efficient OR sustainab* AND value* OR identit* OR knowledge OR belief* OR attitude* OR norm* OR intention* OR behavi*or* OR lifestyle* AND individual OR consumer OR personal OR self AND purchase OR household OR practice OR action OR activism OR nature OR ecosystem OR climate OR planet) AND TITLE (environment* OR ecologic* OR green OR environmental OR pro-environmental OR resource-efficient OR sustainab* AND value* OR identit* OR knowledge OR belief* OR attitude* OR norm* OR intention* OR behavi*or* OR lifestyle*) AND (concept OR conceptualization OR construct OR operationalization) SUBJAREA ( ARTS OR BUSI OR DECI OR ECON OR PSYC OR SOCI )) AND ( LIMIT-TO ( LANGUAGE,"English" ) AND ( LIMIT-TO ( SRCTYPE,"j" ) AND ( LIMIT-TO ( DOCTYPE,"ar" ) OR LIMIT-TO ( DOCTYPE,"re" )  *indicates open end of the word to identify plurals and singular  ^1^ Furthermore results from journals were excluded, that were not related to the subject area or had different foci, or low ranking in the Scimago Journal & Country Rank in the relevant subject areas, respectively. |
|  | *Web of Science*  Example for a combined title-abstract-keyword and topic search (document types: article, indexes scio-expanded, ssci timespan=all years, language; English)  Search 1: (TI=( environment* OR ecologic* OR green OR environmental OR pro-environmental OR resource-efficient OR sustainab*) OR AB= ( list of terms) OR KP= (list of terms)  Search 2 TI=( value* OR identit* OR knowledge OR belief* OR attitude* OR norm* OR intention* OR behavi*or* OR lifestyle*) OR AB= ( list of terms) OR KP= (list of terms)  Search 3: TS=( purchase OR household OR practice OR action OR activism OR nature OR ecosystem OR climate OR planet)  Search 4: TS=( individual OR consumer OR personal OR self)  Search 5: (concept OR conceptualization OR construct OR operationalization)  Search 6: TS1 AND TS2 AND TS3 AND TS4 AND TS5 |
|  | *Google scholar*  Example for direct phrase search: “pro-environmental intention” |
