# Supplementary material for: Concepts Describing and Assessing Individuals’ Environmental Sustainability: An Integrative Review and Taxonomy
Source: Front Psychol. 2022 Jan 5;12:770470. doi: 10.3389/fpsyg.2021.770470 (PMC8766306; doi:10.3389/fpsyg.2021.770470)
Supplement: Supplementary file 3 [file Table_2.docx]

**Table A2** *List of focal constructs and synonymous terms and constructs*

| Construct | Synonyms |
| --- | --- |
| 1 Biospheric values (Stern et al., 1993) | - Biospheric personal values (Bouman et al., 2020) |
| 5 Connectedness to nature (Mayer & Frantz, 2004) | - Emotional affinity towards nature (Kals, Schumacher,& Montada, 1999) (Construct) - Inclusion of nature in self (INS) (Schultz, 2002) (Construct) - Connectivity to nature/ Environmental connectivity (Dutcher et al., 2007) - Identification with nature (Schmitt et al., 2019) |
| 8 Environmental self-identity (Van der Werff et al., 2013) | - Pro-environmental self-identity (Dermody et al., 2018) - Environmentalist identity (Kashima et al., 2014) - Green self-identity (Lalot et al., 2019) |
| 12 Ecological worldview (R. E. Dunlap et al., 2000) | - New environmental paradigm (NEP) (R. Dunlap & Van Liere, 1978) - New Ecological Paradigm (R. E. Dunlap et al., 2000) |
| 14 Ascription of responsibility (Stern et al., 1995) | - Perceived responsibility for environmental damage (Peloza et al., 2013) |
| 15 Climate change risk perception (van der Linden, 2015) | - Climate change belief (Brick, Sherman, & Kim, 2017) - Concern about climate change (Tobler et al., 2012) - Climate concern (Alcock et al., 2017) - Concern for climate (Zhu et al., 2020) |
| 16b Environmental attitude (P. W. Schultz et al., 2004) | - Pro environmental attitude (Lavelle et al., 2015) ( |
| 18 Personal pro-environmental norms (Stern et al., 1999) | - Ecological citizenship (Seyfang, 2005) (construct) - Sustainability citizenship (Barry, 2005) (construct) |
| 22 Green purchase intention (Chang & Wu, 2015) | - Purchase intention for environmentally sustainable products (PI) (Kumar et al., 2017) (construct) |
| 23 Ecological behavior (Kaiser et al., 1999) | - Pro-environmental behavior (Bamberg & Möser, 2007) (construct) - Conservation behavior (Kaiser & Wilson, 2004) (construct) - Environmentally-friendly behavior (Liobikiene & Juknys, 2016) - Environmentally responsible behavior (Thøgersen, 2004) - Environmental behavior (Steg & Vlek, 2009) |
| 24 Environmentalism (Stern, 2000) | - Environmentally significant consumer behavior (Stern, 2000) (Gatersleben et al., 2002) - Non-activism support for the environmental movement (Stern et al., 1999) |
| 25 Sustainable consumption behavior (Geiger et al., 2018) | - Sustainable consumer behavior (Trudel, 2018) |
| 27 Sustainable lifestyles (CSD, 2004) | - Ecological lifestyles (Arnold et al., 2018) (construct) - Green lifestyles (Lorenzen, 2012) - Pro-environmental behaviors (Gatersleben et al., 2010) - Sustainable development lifestyle (Starcic et al., 2018) |
| 28 Ecologically conscious consumer behavior (Roberts & Bacon, 1997) | - Green purchase behavior (Chan, 2001) - Responsible consumer behavior (Buerke et al., 2017) (construct) - Green product consumer choice behavior (Lin & Huang, 2012) |
| 32 Environmental citizenship (Takahashi et al., 2017) | - Green citizenship (Dean, 2001) - Environmental citizenship behavior (Song et al., 2019) - Pro-environmental activist behavior (Schmitt et al., 2019) - Environmental activism (Lee, Lee, Ma, & Cheung, 2019; Steg et al., 2011) |
| 34 Environmental consciousness (Schlegelmilch et al., 1996) | - Ecological concern (Bohlen et al., 1993) |

Note: Numbers of the focal constructs corresponds to numbering in the taxonomy

**References (for Table 2)**

Alcock, I., White, M. P., Taylor, T., Coldwell, D. F., Gribble, M. O., Evans, K. L., Corner, A., Vardoulakis, S., & Fleming, L. E. (2017). ‘Green’ on the ground but not in the air: Pro-environmental attitudes are related to household behaviours but not discretionary air travel. *Global Environmental Change*, *42*, 136–147. https://doi.org/10.1016/j.gloenvcha.2016.11.005

Arnold, O., Kibbe, A., Hartig, T., & Kaiser, F. G. (2018). Capturing the Environmental Impact of Individual Lifestyles: Evidence of the Criterion Validity of the General Ecological Behavior Scale. *Environment and Behavior*, *50*(3), 350–372. https://doi.org/10.1177/0013916517701796

Bamberg, S., & Möser, G. (2007). Twenty years after Hines, Hungerford, and Tomera: A new meta-analysis of psycho-social determinants of pro-environmental behaviour. *Journal of Environmental Psychology*, *27*(1), 14–25. https://doi.org/10.1016/j.jenvp.2006.12.002

Barry, J. (2005). *Resistance Is Fertile: From Environmental to Sustainability Citizenship*. *February*.

Bohlen, G., Schlegelmilch, B. B., & Diamantopoulos, A. (1993). Measuring ecological concern: A multi-construct perspective. *Journal of Marketing Management*, *9*(4), 415–430. https://doi.org/10.1080/0267257X.1993.9964250

Bouman, T., Steg, L., & Johnson-Zawadzki, S. (2020). The value of what others value: When perceived biospheric group values influence individuals’ pro-environmental engagement. *Journal of Environmental Psychology*, *71*, 101470. https://doi.org/10.1016/j.jenvp.2020.101470

Brick, C., Sherman, D. K., & Kim, H. S. (2017). “Green to be seen” and “brown to keep down”: Visibility moderates the effect of identity on pro-environmental behavior. *Journal of Environmental Psychology*, *51*, 226–238. https://doi.org/10.1016/j.jenvp.2017.04.004

Buerke, A., Straatmann, T., Lin-Hi, N., & Müller, K. (2017). Consumer awareness and sustainability-focused value orientation as motivating factors of responsible consumer behavior. *Review of Managerial Science*, *11*(4), 959–991. https://doi.org/10.1007/s11846-016-0211-2

Chan, R. Y. K. (2001). Determinants of Chinese Consumers ’ Green Purchase Behavior. *Psychology and Marketing*, *18*(April 2001), 389–413.

Chang, M.-C., & Wu, C.-C. (2015). The effect of message framing on pro-environmental behavior intentions. *British Food Journal*, *117*(1), 339–357. https://doi.org/10.1108/BFJ-09-2013-0247

CSD. (2004). *Every little bit helps…” Overcoming the challenges to researching, promoting and implementing sustainable lifestyles*.

Dean, H. (2001). Green citizenship. *Social Policy and Administration*, *35*(5), 490–505. https://doi.org/10.1111/1467-9515.t01-1-00249

Dermody, J., Koenig-Lewis, N., Zhao, A. L., & Hanmer-Lloyd, S. (2018). Appraising the influence of pro-environmental self-identity on sustainable consumption buying and curtailment in emerging markets: Evidence from China and Poland. *Journal of Business Research*, *86*, 333–343. https://doi.org/10.1016/j.jbusres.2017.09.041

Dunlap, R. E., Van Liere, K. D., Mertig, A. G., & Jones, R. E. (2000). New Trends in Measuring Environmental Attitudes: Measuring Endorsement of the New Ecological Paradigm: A Revised NEP Scale. *Journal of Social Issues*, *56*(3), 425–442. https://doi.org/10.1111/0022-4537.00176

Dunlap, R., & Van Liere, K. (1978). The “New Environmental Paradigm.” *The Journal of Environmental Education*, *9*(4), 10–19. https://doi.org/10.1080/00958964.1978.10801875

Dutcher, D. D., Finley, J. C., Luloff, A. E., & Johnson, J. B. (2007). Connectivity With Nature as a Measure of Environmental Values. *Environment and Behavior*, *39*(4), 474–493. https://doi.org/10.1177/0013916506298794

Gatersleben, B., Steg, L., & Vlek, C. (2002). Measurement and determinants of environmentally significant consumer behavior. *Environment and Behavior*, *34*(3), 335–362. https://doi.org/10.1177/0013916502034003004

Gatersleben, B., White, E., Abrahamse, W., Jackson, T., & Uzzell, D. (2010). Values and sustainable lifestyles. *Architectural Science Review*, *53*(1), 37–50. https://doi.org/10.3763/asre.2009.0101

Geiger, S. M., Fischer, D., & Schrader, U. (2018). Measuring What Matters in Sustainable Consumption: An Integrative Framework for the Selection of Relevant Behaviors Introduction: The Necessity and Difficulty of Measuring the Sustainability of Consumption Behavior. *Sustainable Development*, *26*, 18–33. https://doi.org/10.1002/sd.1688

Kaiser, F. G., & Wilson, M. (2004). Goal-directed conservation behavior: The specific composition of a general performance. *Personality and Individual Differences*, *36*(7), 1531–1544. https://doi.org/10.1016/j.paid.2003.06.003

Kaiser, F. G., Wölfing, S., & Fuhrer, U. (1999). Environmental Attitude and Ecolgocial Behaviour. *Journal of Environmental Psychology*, *19*, 1–19.

Kals, E., Schumacher, D., & Montada, L. (1999). Emotional affinity toward nature as a motivational basis to protect nature. *Environment and Behavior*, *31*(2), 178–202. https://doi.org/10.1177/00139169921972056

Kashima, Y., Paladino, A., & Margetts, E. A. (2014). Environmentalist identity and environmental striving. *Journal of Environmental Psychology*, *38*, 64–75. https://doi.org/10.1016/j.jenvp.2013.12.014

Kumar, B., Manrai, A. K., & Manrai, L. A. (2017). Purchasing behaviour for environmentally sustainable products: A conceptual framework and empirical study. *Journal of Retailing and Consumer Services*, *34*(February 2016), 1–9. https://doi.org/10.1016/j.jretconser.2016.09.004

Lalot, F., Quiamzade, A., Falomir-Pichastor, J. M., & Gollwitzer, P. M. (2019). When does self-identity predict intention to act green? A self-completion account relying on past behaviour and majority-minority support for pro-environmental values. *Journal of Environmental Psychology*, *61*(January), 79–92. https://doi.org/10.1016/j.jenvp.2019.01.002

Lavelle, M. J., Rau, H., & Fahy, F. (2015). Different shades of green? Unpacking habitual and occasional pro-environmental behavior. *Global Environmental Change*, *35*, 368–378. https://doi.org/10.1016/j.gloenvcha.2015.09.021

Lee, K. M. Y., Lee, J. C. K., Ma, A. T. H., & Cheung, L. T. O. (2019). Does human rights awareness spur environmental activism? Hong Kong’s ‘country park’ controversy. *Land Use Policy*, *87*(January), 104033. https://doi.org/10.1016/j.landusepol.2019.104033

Lin, P.-C., & Huang, Y.-H. (2012). The influence factors on choice behavior regarding green products based on the theory of consumption values. *Journal of Cleaner Production*, *22*(1), 11–18. https://doi.org/10.1016/j.jclepro.2011.10.002

Liobikiene, G., & Juknys, R. (2016). The role of values, environmental risk perception, awareness of consequences, and willingness to assume responsibility for environmentally-friendly behaviour: The Lithuanian case. *Journal of Cleaner Production*, *112*, 3413–3422. https://doi.org/10.1016/j.jclepro.2015.10.049

Lorenzen, J. A. (2012). Going Green: The Process of Lifestyle Change1. *Sociological Forum*, *27*(1), 94–116. https://doi.org/10.1111/j.1573-7861.2011.01303.x

Mayer, F. S., & Frantz, C. M. P. (2004). The connectedness to nature scale: A measure of individuals’ feeling in community with nature. *Journal of Environmental Psychology*, *24*(4), 503–515. https://doi.org/10.1016/j.jenvp.2004.10.001

Peloza, J., White, K., & Shang, J. (2013). Good and Guilt-Free: The Role of Self-Accountability in Influencing Preferences for Products with Ethical Attributes. *Journal of Marketing*, *77*(1), 104–119. https://doi.org/10.1509/jm.11.0454

Roberts, J. A., & Bacon, D. R. (1997). Exploring the Subtle Relationships between Environmental Concern and Ecologically Conscious Consumer Behavior. *Journal of Business Research*.

Schlegelmilch, B. B., Bohlen, G. M., & Diamantopoulos, A. (1996). The link between green purchasing decisions and measures of environmental consciousness. *European Journal of Marketing*, *30*(5), 35–55. https://doi.org/10.1108/03090569610118740

Schmitt, M. T., Mackay, C. M. L., Droogendyk, L. M., & Payne, D. (2019). What predicts environmental activism? The roles of identification with nature and politicized environmental identity. *Journal of Environmental Psychology*, *61*(November 2018), 20–29. https://doi.org/10.1016/j.jenvp.2018.11.003

Schultz, P. W., Shriver, C., Tabanico, J. J., & Khazian, A. M. (2004). Implicit connections with nature. *Journal of Environmental Psychology*, *24*(1), 31–42. https://doi.org/10.1016/S0272-4944(03)00022-7

Schultz, W. P. (2002). Inclusion with Nature: The psychology of human-nature relations. In P. Schmuck & W. P. Schultz (Eds.), *Psychology of Sustainable Development*. Kluwer Academic Publishers. https://doi.org/10.1007/978-1-4615-0995-0

Seyfang, G. (2005). Shopping for sustainability: Can sustainable consumption promote ecological citizenship? *Environmental Politics*, *14*(2), 290–306. https://doi.org/10.1080/09644010500055209

Song, Z., Daryanto, A., & Soopramanien, D. (2019). Place attachment, trust and mobility: Three-way interaction effect on urban residents’ environmental citizenship behaviour. *Journal of Business Research*, *105*(January 2018), 168–177. https://doi.org/10.1016/j.jbusres.2019.08.001

Starcic, A. I., Terlevic, M., Lin, L., & Lebenicnik, M. (2018). Designing learning for sustainable development: Digital practices as boundary crossers and predictors of sustainable lifestyles. *Sustainability (Switzerland)*, *10*(6). https://doi.org/10.3390/su10062030

Steg, L., De Groot, J. I. M., Dreijerink, L., Abrahamse, W., & Siero, F. (2011). General Antecedents of Personal Norms, Policy Acceptability, and Intentions: The Role of Values, Worldviews, and Environmental Concern. *Society & Natural Resources*, *24*(4), 349–367. https://doi.org/10.1080/08941920903214116

Steg, L., & Vlek, C. (2009). Encouraging pro-environmental behaviour: An integrative review and research agenda. *Journal of Environmental Psychology*, *29*(3), 309–317. https://doi.org/10.1016/j.jenvp.2008.10.004

Stern, P. C. (2000). Toward a Coherent Theory of Environmentally Significant Behavior. *Journal of Social Issues*, *56*(3), 407–424. http://web.stanford.edu/~kcarmel/CC_BehavChange_Course/readings/Stern_metareview_2000.pdf

Stern, P. C., Dietz, T., Abel, T., Guagnano, G. A., & Kalof, L. (1999). A value-belief-norm theory of support for social movements: The case of environmentalism. *Human Ecology Review*, *6*(2), 81–97. https://humanecologyreview.org/pastissues/her62/62sternetal.pdf

Stern, P. C., Dietz, T., & Kalof, L. (1993). Value Orientations, Gender, and Environmental Concern. *Environment and Behavior*, *25*(5), 322–348. https://doi.org/10.1177/0013916593255002

Stern, P. C., Kalof, L., Dietz, T., & Guagnano, G. A. (1995). Values, Beliefs, and Proenvironmental Action: Attitude Formation Toward Emergent Attitude Objects. *Journal of Applied Social Psychology*, *25*(18), 1611–1636. https://doi.org/10.1111/j.1559-1816.1995.tb02636.x

Takahashi, B., Tandoc, E. C., Duan, R., & Van Witsen, A. (2017). Revisiting Environmental Citizenship: The Role of Information Capital and Media Use. *Environment and Behavior*, *49*(2), 111–135. https://doi.org/10.1177/0013916515620892

Thøgersen, J. (2004). A cognitive dissonance interpretation of consistencies and inconsistencies in environmentally responsible behavior. *Journal of Environmental Psychology*, *24*(1), 93–103. https://doi.org/10.1016/S0272-4944(03)00039-2

Tobler, C., Visschers, V. H. M., & Siegrist, M. (2012). Consumers’ knowledge about climate change. *Climatic Change*, *114*(2), 189–209. https://doi.org/10.1007/s10584-011-0393-1

Trudel, R. (2018). Sustainable consumer behavior. *Consumer Psychology Review*, *October 2018*, 85–96. https://doi.org/10.1002/arcp.1045

van der Linden, S. (2015). The social-psychological determinants of climate change risk perceptions: Towards a comprehensive model. *Journal of Environmental Psychology*, *41*, 112–124. https://doi.org/10.1016/j.jenvp.2014.11.012

Van der Werff, E., Steg, L., & Keizer, K. (2013). It is a moral issue: The relationship between environmental self-identity, obligation-based intrinsic motivation and pro-environmental behaviour. *Global Environmental Change*, *23*(5), 1258–1265. https://doi.org/10.1016/j.gloenvcha.2013.07.018

Zhu, J., Hu, S., Wang, J., & Zheng, X. (2020). Future orientation promotes climate concern and mitigation. *Journal of Cleaner Production*, *262*, 121212. https://doi.org/10.1016/j.jclepro.2020.121212
